# Sulfonated Pentablock Copolymer (Nexar^TM^) for Water Remediation and Other Applications

**DOI:** 10.3390/polym16142009

**Published:** 2024-07-13

**Authors:** Simona Filice, Viviana Scuderi, Silvia Scalese

**Affiliations:** Consiglio Nazionale delle Ricerche, Istituto per la Microelettronica e Microsistemi (CNR-IMM), Ottava Strada n.5, 95121 Catania, Italy; viviana.scuderi@imm.cnr.it

**Keywords:** Nexar^TM^, sulfonated copolymer, water, energy, coating, filtration, photocatalysis, adsorption, antibiofouling

## Abstract

This review focuses on the use of a sulfonated pentablock copolymer commercialized as Nexar^TM^ in water purification applications. The properties and the use of sulfonated copolymers, in general, and of Nexar^TM^, in particular, are described within a brief reference focusing on the problem of different water contaminants, purification technologies, and the use of nanomaterials and nanocomposites for water treatment. In addition to desalination and pervaporation processes, adsorption and photocatalytic processes are also considered here. The reported results confirm the possibility of using Nexar^TM^ as a matrix for embedded nanoparticles, exploiting their performance in adsorption and photocatalytic processes and preventing their dispersion in the environment. Furthermore, the reported antimicrobial and antibiofouling properties of Nexar^TM^ make it a promising material for achieving active coatings that are able to enhance commercial filter lifetime and performance. The coated filters show selective and efficient removal of cationic contaminants in filtration processes, which is not observed with a bare commercial filter. The UV surface treatment and/or the addition of nanostructures such as graphene oxide (GO) flakes confer Nexar^TM^ with coating additional functionalities and activity. Finally, other application fields of this polymer are reported, i.e., energy and/or gas separation, suggesting its possible use as an efficient and economical alternative to the more well-known Nafion polymer.

## 1. Introduction

Water purification is one of the most important research areas in the world since clean water is fundamental for life and many human activities, but its abundance continues to decrease because of the dramatic effects of climate change. Furthermore, water quality is negatively affected by an increase in pollution in oceans, rivers, lakes, and other water sources. This pollution is generated by widespread civilization as well as industrialization processes. For these reasons, the limited availability and the low quality of freshwater are serious concerns today, even if water occupies more than 70% of the earth’s crust [1,2,3,4]. According to the World Health Organization and UNICEF report released in June 2019, one out of every three people around the world lacks safe drinking water access [5,6]. The 2021 report of the European Environment Agency affirmed that water stress affects 30% of the population in Europe. Water stress occurs when water of sufficient quality is not abundant enough to meet people’s demands. This situation is going to become worse with the climate crisis [7]. The trends are especially worrying for southern and southwestern Europe, where river discharge during summer could decline by up to 40% consequently leading to a 3 °C temperature rise. In those areas, agriculture, public water supply, and tourism put the main pressure on water availability, with a significant seasonal peak in summer.

Clean freshwater is fundamental for the development and maintenance of the eco-systems on which all livelihoods rely: waterborne diseases are brought on by a decline in the condition of aquatic systems, influencing the lives of countless individuals worldwide and eventually resulting in death [8]. Furthermore, clean water is also important for the socio-economic development of countries because it is required for drinking, sanitation, agricultural activities, and industry [9]. Low water availability negatively affects the industrial and social development of both developing and industrialized nations: a lack of water reduces energy and food production, environment quality, economic development, and the health of the population. For example, in developing countries, many women do not attend school or hold a job since they must walk 3–4 h per day in order to collect and carry back water home. In this way, their family income is reduced, and they do not have instruction and/or economic independence.

The last Water Development Report 2024 from the United Nations drew attention to the complex and interlinked relationships between water, prosperity, and peace, describing how progress in one dimension can have positive, often essential, repercussions on others [10]. Between 2022 and 2021, half of the global population (about 1.4 billion people) is facing grave water shortages, causing the death of nearly 21,000 individuals. Furthermore, water influences the economy in many ways; for example, 50% of jobs in rich countries depend on water, and this value increases to 80% for the lowest-income countries. In March 2023, UNESCO warned of the impending risk of a global water crisis [11]. In this scenario, constant attempts were devoted to enhancing the current technology and making innovations in order to offer cost- and energy-efficient remediation methods. In order to find the best methodology for water purification, it is fundamental to consider that water is a really complex research field, and no one solution fitting all problems concerning it is possible. Water is a complex matrix: many different contaminants could be present in a water sample depending on its origin and pathway. Table 1 reports the different water pollutant classes. 

Organic pollutants can be generated from natural or industrial sources and are toxic and cancerogenic for human life. Furthermore, the presence of NOM and other organic waste could impart color to water, reducing its aesthetic qualities [12]. Furthermore, organic matter could reduce the efficiency of water remediation technologies by reducing the efficiency and lifespan of filters used in many processes (i.e., membrane fouling) by competing with other pollutants for adsorption sites or through the formation of toxic by-products [13]. 

Similarly, heavy and radioactive metal ions could originate from anthropogenic sources or geological phenomena, and these are highly toxic for humans because of their bioaccumulation in the human body by the food chain. 

Microorganisms are the third contaminant class and have a devasting effect on public health and the socio-economic development of developing countries. New efficient methods or materials for disinfecting water from traditional and emerging pathogens are highly desirable, and these methods/materials should avoid the introduction of toxic by-products, which occur by using halogenated compounds [14,15]. 

Microorganisms that are not commonly monitored in the environment within synthetic or naturally occurring chemicals constitute a new class of emerging contaminants [16]. These have the potential to contaminate surface water, wastewater, and groundwater sources and bioaccumulate in the human body, causing deleterious effects on endocrine systems and thyroid gland, infertility, and cancer. 

**Figure 1:**
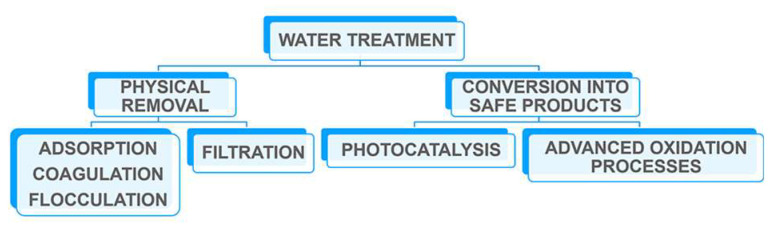
Scheme of water treatment methodologies.

For each contaminant, different removal methodologies are required, and the possible interactions between contaminants should be considered since this could reduce the efficiency of removal technologies or induce the formation of other toxic by-products. In addition, the chosen methodology for water remediation should be fitted within the economic sources available in the country where dirty water is present. These goals present exciting opportunities for the research community. Looking at existing technologies for water and wastewater treatment, the most common methods are reported in the scheme of Figure 1 and briefly described in the following paragraphs.

Water contaminants could be physically removed from water, concentrated, and/or converted to safer products. Often, these different remediation techniques are used in sequence for the removal/degradation of different water contaminants. The removal of water contaminants such as organic and inorganic compounds and microorganisms could occur through an adsorption process and filtration technology. In this regard, it was shown that polymeric materials such as electrospun nanofibers, membranes, porous films, hydrogels, and nanocomposites are protagonists in these decontamination processes [17,18,19]: in particular, polymeric membranes have recently found large applications because of their properties, i.e., easy pore-formation, high mechanical flexibility, and low cost.

The main concern in designing membrane adsorbents or filters is obtaining permeable membranes with a high adsorption capacity; in other words, it is always necessary to combine both the properties of selectivity and high flux so as to enrich the efficiency of separation [20]. In this regard, nanoporous membranes made by block copolymers meet the desired requirements for controlled separation because of their tunability in terms of pore dimensions and distributions within the possibility of being selectively functionalized [21]. Block copolymers are composed of macromolecules, and the repeating units are covalently connected for long-range; in this way, the resulting molecules have ordered structures with nanoscopic heterogeneity while maintaining the physical properties of each component. These properties allow the block copolymers to interact with contaminants both physically and chemically [22,23,24,25]. Furthermore, functional groups such as carboxylic acids, amines, hydroxyl groups, or sulfonic acids have a great affinity for pollutants, forming stable complexes with them. In particular, sulfonation is a substitution or addition chemical reaction used to incorporate sulfonic acid groups (SO_3_H) in a polymer chain by a chemical bond to a carbon atom [26]. This is one of the most employed techniques for obtaining amphiphilic or hydrophilic block copolymers by introducing a polar functional group into one segment of the block copolymer. The introduction of sulfonic groups improves the ionic conductivity, hydrophilicity, and solubility in polar solvents for the solution processability of polymeric chains. Sulfonic groups are active and selective sites for contaminants adsorption [27,28,29]; these could positively take part in the photocatalytic degradation of water pollutants [28,29,30,31], and these have been recently investigated for antimicrobial and antibiofouling activity [32,33]. Indeed, Peddinti [34] recently reported that midblock-sulfonated multiblock polymers constitute a facile, inexpensive, and environmentally benign way to avoid the proliferation of microbes: this polymer provided hydrophilic and self-sterilizing surfaces against a wide range of Gram-positive and Gram-negative bacteria, three of which are antibiotic-resistant and these surfaces are equally effective against infectious virus strains. The shown activity depends on the degree of midblock sulfonation, which is ascribed to the acidic character of sulfonic groups. Furthermore, sulfonic group acidity, i.e., their antimicrobic activity, can be fully rejuvenated to its maximum performance level by relatively short immersion in acidic solutions. 

This review focuses on the use of a sulfonated pentablock copolymer (s-PBC), commercially named Nexar^TM^ and produced by Kraton LLC, in water treatment alone or as a matrix for embedded nanomaterials. Then, the s-PBC properties and reasons for its use will be described, and several examples of its application for the removal of contaminants (i.e., microorganisms, organic molecules, and heavy metals) by different methodologies (such as adsorption, filtration, and photocatalysis processes) will be reported. 

The investigated materials combine the advantages of nanomaterials and polymer technologies to physically remove and/or degrade water contaminants. Nexar^TM^ polymer, for its particular molecular architecture and the presence of sulfonic groups, is shown to be efficient, low-cost, and green when used as an adsorbent, filter, or support for photocatalytic particles in water remediation technologies. Finally, the possible use of this polymer in different research areas is reported, showing that it is an efficient and low-cost alternative to existing well-known polymers such as Nafion.

## 2. Sulfonated Block-Copolymer for Water Purification

The dominant materials for water purification membranes are polymers because of their low cost, facility to control the resulting film structures, and scalable production capacity, but mainly because of their satisfying performances. New functionalities to membrane separation have been induced by nanotechnology, i.e., the embedding of nanoparticles within polymeric layers or the structurization of polymeric layers at the nanoscale. 

Membranes for filtration are typically made from polyacrylonitrile (PAN), polyvinylidene fluoride (PVDF), polyethersulfone (PES), and polysulfone (PSF). Their main disadvantages are membrane fouling, low chlorine resistance, low long-term stability, etc. For water treatment, polymeric membranes should have a good tradeoff between hydrophobicity and hydrophilicity since the former ensures mechanical, chemical, and thermal stability while the latter characteristic ensures high water flux, high rejection ratio, and low operation pressure. The low cost, recyclability, environmentally benign, and energy-saving features are also attractive advantages of a polymeric material, making it a good candidate for future drinking water purification. To achieve a good tradeoff between permeability and selectivity and eliminating fouling, engineering of new advanced materials (i.e., polymeric, inorganic, block copolymers, and nanocomposite membrane) is needed and new strategies to optimize morphology and structure of just known membranes have been investigated [35]. 

**Figure 2:**
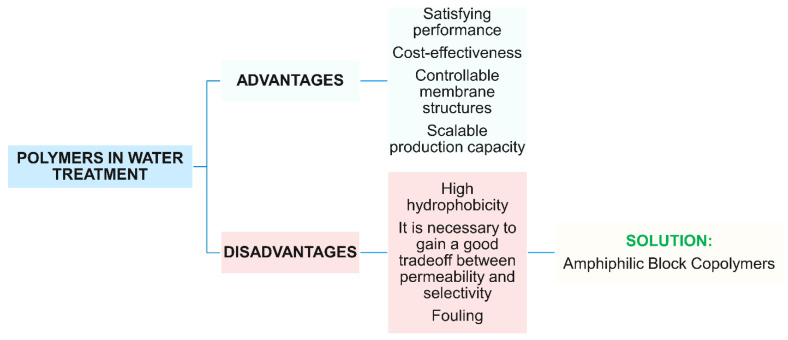
Scheme of advantages and disadvantages of using polymers in water treatment.

Figure 2 reports a scheme of the main advantages and disadvantages of polymeric materials in water treatment and relative solutions. This scheme is fully discussed in the following paragraphs.

Starting from the results of Lyman [36,37] reporting for the first time the use of a block copolymer (BCP) in blood dialysis, BCPs represent the main protagonist in the field of water applications because of their excellent pore size tunability and narrow distributions, but in particular, for their excellent control in terms of functionalization, permeability, thickness, and fluidity. Compared with common organic and inorganic membranes, BCP membranes ensure thermal, mechanical, and chemical stability and long life [38]. Most accessible BCP membranes have pore sizes in the range of 5–50 nm, which belong to the category of ultrafiltration membranes [39]. Recent progress has expanded their applications from ultrafiltration (UF) to nanofiltration (NF) and reverse osmosis (RO) processes via various strategies, such as BCP molecular design [40] and post-modification [41,42]. 

As recently reported by [43], a block copolymer (BCP) is a macromolecule composed of at least two different homopolymers organized in blocks of different chemical composition: identical monomer units repeat in each block, and different polymer segments are covalently bonded. This pattern of covalent bonds ensures chemical, mechanical, and thermal stability. Furthermore, the chemical composition of blocks and their immiscibility generate a phase separation at the microscopic scale, known as microphase separation [44].

This microphase segregation results in BCPs self-assembling into domains with periodicities varying in the order of the nanometers (1–100 nm). Thanks to BCP architecture, the properties of selectivity and high flux are combined in order to enrich the efficiency of separation. BCPs are necessary for the fabrication of membranes and can be made by the controlled radical polymerization process (CRP), which is well-known for fabricating block copolymers with different morphologies such as linear, star-shaped, etc. [45]. Indeed, the main advantage of block copolymers is the possibility of separating them into many different structures, such as cubic, cylindrical, bicontinuous cubic, and lamellar structures. Cylindrical structures usually work with the cylindrical pores aligned perpendicularly and continue to the film surface, while bicontinuous cubic structures can offer interconnected pores all over the membrane with accordingly higher hydraulic resistance. Since BCP membranes are generated from solution, their final structure and geometry could be tuned, for example, by changing the dispersing medium, the chain length of individual components, composition, and Florry–Huggins Parameter [46,47]. The major strategies for BCP membrane preparation include film casting followed by solvent evaporation and self-assembly with non-solvent-induced phase separation (SNIPS) [48]. The latter allows for induced pore formation and the adjustment of pore size. 

Block copolymers could interact physically and chemically with contaminants in water, and their selectivity is affected by their micellar structures and nanoscale architectures. The last ones are tunable according to the composition and molecular weight of the constituent monomers [49]. 

Cooney studied the diffusion of water through a polystyrene-b-polylactideme (PS-PLA) based di-block copolymer nanoporous monolith [50]. 

Higher separation as well as permeability than in currently available membranes were obtained by ABC triblock terpolymers containing polylactide, poly(dimethylacrylamide), and polystyrene, which could self-assemble as aligned cylinders [51]: these polymers showed a larger hydraulic permeability and sharper molecular weight cut-off. Similarly, a desirable and controlled porosity was induced in the polymerization of dicyclopentadiene during the membrane formation process using a polylactide–polynorbornenylethylstyrene block polymer structural template: the pore structure was formed by etching the polylactide component with dilute aqueous base [52].

These membranes were effective for ultrafiltration, with molecular weight cutoffs (MWCOs) consistent with theoretical predictions and tunable according to the size of the constituent blocks in the templating copolymer. Wander modified cellulose membranes with poly(N-isopropylacrylamide)-block-poly([polyethyleneglycol] methacrylate) nanolayers resulting in a unique porous morphology that confers the material with excellent purification and antifouling properties [53]. Starting from [54] on a pH-responsive BCP membrane, Nunes developed a switched pH-responsive PS-b-P4VP polymeric membrane by the assembly of BCPs in the presence of metal followed by non-solvent (water) induced phase separation, and this resulted in uniform polymeric nanochannels with a diameter in the sub 10 mm range and height in the 400 nm range [55]. 

Phillip fabricated a nanoporous membrane containing 24 nm diameter monodisperse pores by using poly (styrene-b-lactide), and the membranes showed an enhanced ability to refuse dissolved solutes [56]. Augustina used the self-assembly of the amphiphilic BCP for the synthesis of porous nanoobjects with different morphologies controlled by the pores and transmembrane pressure [57]. 

An enhancement in hydrophilicity, permeability, and fouling resistance of PSF membranes was observed as an effect of the surface topology [58].

Excellent antifouling properties were recently obtained by [59] using a BCP membrane over PVDF microfilter membranes.

BCPs are good candidates for oil–water separation because of their amphiphilic character, i.e., the hydrophilic part could interact with water and separate it from the oil concentrated in the hydrophobic portions. Furthermore, BCP membranes have better separation efficiency, flux, and anti-fouling properties. 

Indeed, Rajasekahr obtained higher flux, oil rejection, and higher fouling resistance in comparison with neat PVDF membrane-making blends of PVDF and BCP for separating oil–water emulsions [60].

By adjusting the viscosity of blends through variations in the concentration of each component, it was possible to tune the membrane thickness, affecting the selectivity and the effectiveness of the separation process.

In addition to the advantage of BCPs nanoscale architecture, new functionalities and/or selectivity can be added to them by the presence of other additional functionalities on the copolymer backbone, i.e., in the pores, such as charge separation, selective adsorption, anti-fouling, and chemical conversion. Hence, different techniques such as coating, grafting, blending, and chemical functionalization are commonly employed for incorporating additional functionalities into the pores [38]. The sulfonic acid group is commonly used as a functionality in macroporous or gel-type polymer matrices to create well-known strong acid cation ion-exchange resins. This functionality is acidic and has a specific order of selectivity for cations, influenced by both charge and ionic radius. Indeed, an ion exchange resin was proven to be an effective method to remove heavy metals from wastewater, and the resin can be reused through a regeneration process with sodium chloride [61].

**Figure 3:**
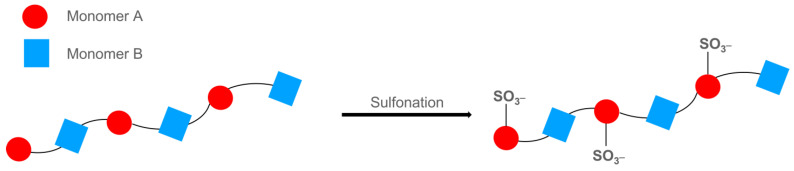
Scheme of selective sulfonation of a block copolymer.

Sulfonation is a chemical process allowing the incorporation of sulfonic acid groups (SO_3_H) on a polymeric chain: this consists of the substitution or addition reaction of a SO_3_H group by bonding to a carbon atom (or rarely to a nitrogen atom) [62]. Figure 3 displays a scheme for the selective sulfonation of a block copolymer.

In general, sulfonation is achieved by a homogeneous reaction in hydrocarbon or chlorinated solvents, with the sulfonation agent (H_2_SO_4_, SO_3_, acyl and alkyl sulfates, and chloro-sulfonic acid) and polymer in separate phases [63]. As reported by [64], the sulfonation process could be conducted before the crosslinking of the polymer, but this limits the sulfonation degree, or sulfonic groups could be added after crosslinking but with low control on the sulfonation yield, resulting in membranes with too high a hydrophilicity. In the first case, at least one of the monomers has the sulfonic groups before the cross-linking; the latter is a post-modification reaction in which, after BCP synthesis, the whole chain is sulfonated at suitable specific positions.

Recently, aromatic polymers have been used as constituents in BCPs because of their strong mechanical properties, high thermal stability, and good chemical resistance. Furthermore, their ionic conductivity, hydrophilicity, and solubility in polar solvents for solution processability could be ameliorated by their sulfonation [65]. Selective sulfonation has been studied on aromatic rings and polydienes after their hydrogenation [66,67]. Polystyrene is the most studied since the sulfonation of this molecule proceeds easily through an electrophilic aromatic substitution [26,43].

Sulfonilic groups are hydrophilic; thus, their presence on the hydrophobic chain confers an amphiphilic character. These polar groups give rise to different chemical interactions such as electrostatic, dipole–dipole, Van der Waals, or hydrogen bonds.

To achieve phase segregation, only one of the copolymer blocks must be modified; that is, sulfonation selectivity is needed.

Concerning block copolymers, the acetyl sulfate complex was used for the first time to modify three-arm star-branched block copolymer ionomers, consisting of butadiene elastomeric inner blocks and oligostyrene ionic outer blocks, and a final sulfonation degree close to 90% was achieved [68]. The mechanical behavior of the obtained material was affected by the counterion used during neutralization.

Sulfonation was used to convert poly(styrene-b-methyl methacrylate) copolymers into ionomers with proton conductivity that made these polymers suitable as roton exchange membranes: the final sulfonation degree of polystyrene block was in the range of 20 to 30%; resulting in an increase in the glass transition temperature [69]. Similarly, Tsang used the same sulfonation agent for the modification of poly([vinylidene difluoride-cohexafluoropropylene]-b-styrene) for application in proton exchange membranes [70]: the ion exchange capacity and size of the membrane ionic aggregates increased with the sulfonation degree. Afterward, Ruiz Colo et al. prepared membranes for fuel cells from a block of poly(styrene-isobutylene-styrene) [71] through sulfonation by the acetyl sulfate complex. Thanks to the ionic interaction between the phosphonate and sulfonated groups, the resulting membrane revealed good performance with respect to Nafion in terms of both higher proton conductivity and lower permeability to methanol crossover. As another source of SO_3_-, Noshay used the sulfur trioxide-triethyl phosphate complex for the first time [72]. Gatsouli used this complex for the modification of poly(sulfonated styrene-b-tert-butylstyrene), and micelles of this copolymer were used for the synthesis of CdS and CdSe nanoparticles [73]. More recently, Politakos et al. synthesized a polystyrene-b-polyisoprene copolymer and gave to this polymer amphiphilic character [74].

Chlorosulfonic acid is another reagent used for the sulfonation of block copolymers: poly(styrene-butadiene-4-vinylpyridine) copolymers were sulfonated after synthesis, and the resulting membrane had substantial cation and anion exchange capabilities [75]. Later, Xu reported the synthesis of sulfonated poly(styrene-b-vinylidene fluoride-b-styrene) triblocks within a degree of post-sulfonation on the order of 10 to 50% [76]. The advantage of the block copolymer structured membranes was reflected in the ionic conductivity, compared with the random copolymer. Yang synthesized linear and star copolymers of polystyrene and poly(4-tert-butylstyrene), and the sulfonation of the PS blocks was carried out selectively by using the sulfur trioxide-triethyl phosphate complex up to 80% [77].

As reported above, one of the main advantages of BCPs is their ability to self-assemble into well-defined nanostructures. Furthermore, the size, shape, and periodicities of domains are easily controllable by variations in experimental parameters, such as the polymerization degree (*N*), the volume fraction (*f*) of each block, and the interaction parameter (χ). In other words, for neutral BCPs, it is possible to predict the resulting nanostructures and morphologies [26]. In contrast, after sulfonation, this predictability is reduced because of the introduction of an amphiphilic character. In this case, many physical interactions between charged and non-charged domains could occur.

Indeed, in sulfonated systems, the copolymer is composed of hydrophilic and hydrophobic segments with lower miscibility between each other and high repulsive energies. Anyway, the chemical composition of a sulfonated copolymer could induce specific hydrophilic channels in the cast films that are fundamental for some applications. For example, Yang observed an enhancement in the phase separation process and the formation of larger hydrophilic channels when the side chains in poly(ether sulfone) multi-block copolymers were grafted with densely pendant sulfoalkoxyl side chains. This modification resulted in a positive impact on the performance of these materials as a proton-conductive membrane [78].

Loveday carried out structural investigations on butadiene-tert-butyl methacrylate and butadiene/styrene-tert-butyl methacrylate after their sulfonation: the initial non-oriented and rod-like morphologies turned into spheroids as a consequence of the interaction with ionic domains. Furthermore, the glass transition temperature of these materials has been shown to be dependent on the length of the ionic segment [79].

**Figure 4:**
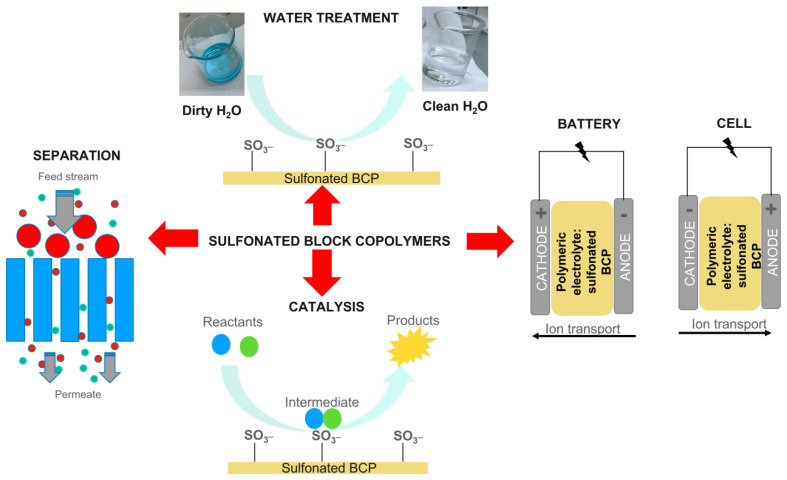
Scheme of the possible use of sulfonated block copolymers.

As shown in Figure 4, sulfonated PBCs are widely used in water treatment as adsorbents and/or filters [80], in catalysis [81], gas separation [82,83], as polyelectrolytes in the proton exchange membrane fuel cell, and the battery industry [84,85,86,87].

In comparison with random copolymers, the self-assembly process of BCPs into ordered nanostructures such as lamellar, gyroid, hexagonal packed cylinders, and body cubic-centered phases enhances the ionic conduction capabilities since H^+^ ions conductivity is favored by the presence of hydrophilic pathway [88]. Indeed, the modification of the nanostructure size, shape, and periodicity of the ionic domain in sulfonated polymers reflects in the control of their conductivity. Starting from this aspect, another interesting research area arises.

For example, [89] studied the structure–property relationship in sulfonated polystyrene–polymethyl methacrylate BCPs varying the sulfonation degree. They observed that the ionic conductivity of membranes increased according to different morphologies that were affected by the degree of sulfonation (i.e., isotropic phase < cylindrical hexagonal phase < hexagonally perforated lamellar phase < lamellar phase). A similar work was reported by [43] for polymethyl methacrylate and polystyrene BCP systems. According to [43], the introduction of sulfonic acid groups induces hydrogen bonds between the charged segments, resulting in different microphase separations, i.e., different morphologies. Mineart et al. [90] studied the self-assembly process of a midblock sulfonated multiblock copolymer according to solvent polarity, aiming to obtain high water and ion transport. Recently, Politakos et al. [74] reported a comparative study on the poly(styrene-b-isoprene) (PS-b-PI) structures obtained by its hydrogenated and sulfonated derivatives. In the first case, well-ordered, hexagonally close-packed cylinders were observed. In contrast, after sulfonation, the membranes cast with cyclohexane showed the formation of horizontal cylinders. This morphology was turned micellar after annealing or using a polar solvent.

The possibility of tuning BCP nanostructures by varying their charge was theoretically demonstrated by [91]: according to their studies, nanostructures such as percolated phases that are not observable in conventional uncharged block copolymers could occur for charged BCPs because of the highly asymmetric charge cohesion effects. These new structures are desired for ion transport and are inaccessible to conventional uncharged block copolymers, including percolated phases desired for ion transport.

Generally, the conductivity properties of sulfonated BCPs as proton exchange membrane materials are directly related to the sulfonation degree: a high sulfonation degree ensures that the hydrophilic clusters increase and the resulting ion channels are effectively connected [88]. Anyway, a high swelling degree due to a high sulfonation degree leads to a dramatic decrease in the dimensional stability of the membrane. Block copolymer architecture could help in tuning the hydrophilic/hydrophobic character of proton exchange membranes inducing selective sulfonation, i.e., controlled sulfonation degree while still maintaining greater stability to swelling with the adsorption of water.

The above-described characteristics are also fundamental for the application of these polymers in water purification: for example, charge densities, water molecular transport rate, and mechanical stability were optimized by a combination of hydrophilic sulfonated polystyrene and cross-linkable hydrophobic hydrogenated isoprene chains [80]. These polymers reportedly possess a high efficiency in removing different ions within higher water permeability and mechanical stability. Chen studied the mechanical and water transport properties of a system of ionomeric block copolymers with utmost interest in water filtration and proton exchange membranes [64]. They showed that by increasing the sulfonation level, the proton conductivity increased within hydrophilicity, but the membranes resulted softer; decreasing the molecular weight of the sulfonated styrene mid-block could avoid membrane softening, but its hydrophobicity increased, causing a shorter lifetime, higher operation cost, and smaller application range.

Sulfonation could also be used on waste plastic; recycled polystyrene cups treated with a sulfonating polymer were used as flocculants in a water treatment process [92]. After adding sulfonated polystyrene (1 mg/L) and ferric chloride (200 mg/L), the turbidity was reduced from 200 NTU to 5.64 NTU. Similarly, (waste plastic was transformed into a cation exchange material by sulfonation [92]: this material showed an ion exchange capacity of 40.85 mg Cr^3+^/g.

## 3. Nexar^TM^ Polymer for Water Remediation

A sulfonated pentablock copolymer (s-PBC), commercialized as Nexar^TM^, that has recently shown up as a promising material (as itself or in combination with nanomaterials) for water purification remediation, is presented in this review.

Characteristics that make it suitable for water purification are discussed in detail. Some examples of its use in adsorption, filtration, and photocatalytic processes for the removal of organic and inorganic contaminants from water are reported. This polymer was used for the preparation of free-standing membranes or as an active coating layer for commercial filters, as shown in the following paragraph. For the last purpose, the polymer’s antimicrobial and antibiofouling properties were reported.

### 3.1. Nexar^TM^ Properties

**Figure 5:**
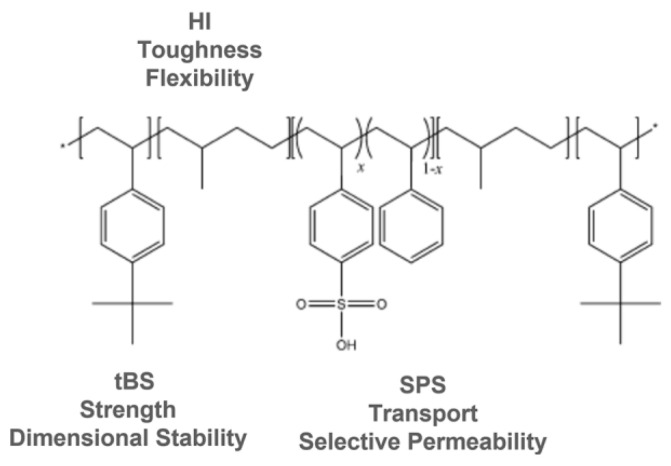
Nexar^TM^ structure as reported in [93].

Nexar^TM^ by Kraton LCC is a symmetric pentablock copolymer comprised of poly[*t*-butyl styrene-*b*-hydrogenated isoprene-*b*-sulfonated styrene-*b*-hydrogenated isoprene-*b*-*t*-butyl styrene] (tBS-HI-SS-HI-tBS), in which the sulfonated midblock provides the ionic character, while the outer blocks provide the flexibility of a low glass transition (*T*_g_) material and the strength of a high *T*_g_ material. The structure of Nexar is reported in Figure 5.

The investigated polymer is prepared using anionic polymerization to synthesize the base block copolymer, catalytic hydrogenation of the residual isoprene C=C bonds, followed by a post-polymerization sulfonation process [94]. The alternation of hydrophobic and hydrophilic domains in this structure makes this polymer stable in water while still absorbing it. The sulfonic groups confer high proton conductivity and water permeability and act as active sites for adsorption [93,94,95,96]. Unlike sulfonated poly(styrene-b-hydrogenated butadiene-b-styrene) or SEBS materials, selective sulfonation is achieved thanks to this specific molecular architecture: the *tert*-butyl group and the polymer backbone protect, respectively, the *para* and *ortho* positions of the phenyl ring avoiding the sulfonation of the *tert*-butyl styrene end blocks. This results in a polymer with controlled swelling and good mechanical properties in the hydrated state, i.e., a good trade-off between hydrophilicity and mechanical stability. The hydrogenated isoprene block gives the copolymer additional toughness [93,94,95,96].

The film structure, physical properties, and transport characteristics are dependent upon the casting solvent, sulfonation level, and processing method [97]: the SAXS profiles reveal a lamellar morphology for the unsulfonated polymer. Indeed, three main peaks close to nq were observed (i.e., *q*_1_ = 0.230 nm^−1^ (*d*_1_ = 27.3 nm) and *q*_2_ = 0.356 nm^−1^ (*d*_2_ = 17.6 nm) and a weak higher-order peak at *q*_3_ = 0.675 nm^−1^ (*d*_3_ = 9.31 nm)). By increasing the sulfonation degree, no long-range order is present because of ion aggregation and domain formation. This was observed in the SAXS profile since the *q_3_* shifts to lower values, and the other two principal peaks merge into a single broad peak. Furthermore, the scattering intensity and average *d*-spacing increased with the sulfonation degree. The lack of order is attributable to the process method and solvent, which revealed the non-equilibrium nature of these materials. The nature and distribution of micelles in Nexar^TM^ solutions depend on the degree of sulfonation and solvent used in the process, and these persist in dried films. This is a key aspect since appropriate processing conditions could be chosen to obtain specific morphologies both in solution and dried film. The structure, morphology, and properties of the resulting membranes are affected by the size and shape of the self-assembled structures. As also confirmed by TEM images, in apolar solvents such as cyclohexane/heptane mixtures, the sulfonated pentablock copolymer solutions form spherical micelles: the hydrophilic part (sulfonated styrene) is the core surrounded by a corona of solvated HI-tBS [98,99]. The dimensions of micelles and their number depend on the sulfonation degree: dimensions increase with an increasing sulfonation degree; in contrast, their number per unit volume decreases. This is ascribed to the incompatibility between ionic (SS) and non-ionic (tBS and HI) blocks. During membrane formation by solvent casting, i.e., solvent evaporation, the spherical micelles compact: the HI-tBS coronae merge to form discrete SS microdomains. These domains are larger and have a higher sulfonation degree, transforming to a bicontinuous morphology with interconnected SS microdomains.

With regard to solvent casting, inverted micelles with exposed sulfonilic groups occur, forming connected ionic domains by using polar solvents [100]: membranes prepared from apolar (i.e., cyclohexane) solution displayed ion-rich spherical microdomains; in contrast, those cast from polar solvents (e.g., tetrahydrofuran) exhibited coexisting nonpolar cylinders and lamellae; thereby providing a continuous pathway through which ions and other polar species can diffuse.

Specific studies on the critical micellar concentration values have not been reported: Choi in [101] reported an estimation of the number of micelles per unit volume (*n*) by the Kinning–Thomas model at a fixed polymer solution concentration (i.e., 11%) and as a function of increasing IEC. In particular, the volume fraction of micelles, calculated from micelle radius and number density, showed an increase and then a plateau with increasing IEC, while the number of micelles per unit volume (*n*) decreased with IEC.

It is possible to predict the morphology of solution-cast Nexar films by screening the initial solution-state structure, which is controlled by tunable polymer–solvent interactions [102]. In this way, specific film morphologies and transport properties could be achieved. The self-assembly in solution was controlled by using mixtures of polar and nonpolar solvents: selective solvents to one block promote self-assembly into highly ordered structures, i.e., lamellar and films cast from these solutions are also lamellar. In contrast, neutral solvents lead to a disordered state and, consequently, disordered cast films. These distinct film morphologies affect its transport properties: passing from ordered lamellae to a disordered network increases both the water uptake and the proton conductivity. Before this study, the same author investigated the structure and properties of Nexar films as a function of “wet-dry cycles” [103]. These cycles favor a structural transition toward increasingly interconnected sulfonated domains affecting positively water and ion transport. However, cycling can also induce mechanical deformations that reduce ductility, swelling, and water uptake. Therefore, the transport properties of these materials result from a balance between the above-mentioned aspects.

Amphiphilic block copolymers for water treatments and electrochemical devices require specific properties in terms of structure, water uptake, and transport, and these are affected by the processing environment in many different ways, so their design represents an interesting challenge for the research community. In the following paragraphs, some examples of the use of Nexar polymer as proton exchange membranes in energy applications or as desalination and pervaporation membranes are shown. With regard to water remediation, a leading role is played by polymeric nanocomposites obtained by the dispersion of nanoparticles inside a polymeric matrix. In this regard, the sulfonic groups in the Nexar structure could interact with nanoparticles, increasing their dispersion, i.e., obtaining more homogenous nanocomposites and/or inducing chemical modifications of nanoparticles [29,31]. Furthermore, the micellar structure of this polymer could play a key role in the self-assembling of nanoparticles in the polymeric layer, determining the final structure at the nanoscopic level of the polymeric nanocomposites. Indeed, the properties of nanocomposites depend on the interaction between the polymeric matrix and the nanofillers.

**Figure 6:**
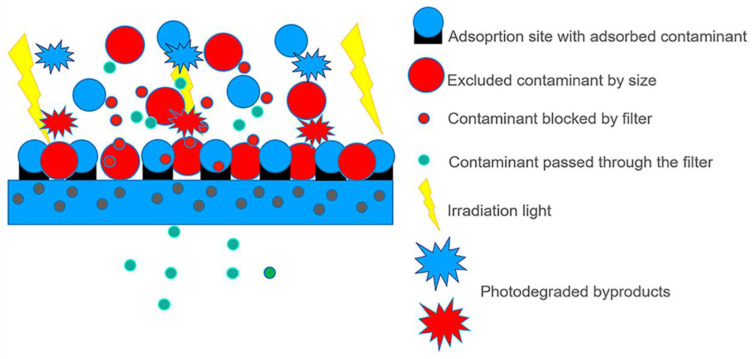
Scheme of all the processes (adsorption, filtration, and photocatalysis) involved in water contaminant removal by Nexar nanocomposites.

Figure 6 is a scheme of all processes that could be involved when a Nexar nanocomposite is used for water purification. The composite is obtained by the dispersion of active nanoparticles inside the polymeric matrix.

In this case, contaminant removal could occur through adsorption, filtration, and photocatalysis if the dispersed nanomaterial has photocatalytic properties.

Adsorption could occur both on active sites present on the surface or through the polymer (i.e., sulfonic groups) and on the nanoparticle’s surface inside the membrane. This system highly increased the surface area, i.e., the number of active sites for adsorption.

In addition to adsorption, contaminants that do not interact with active sites could be removed by filtration according to their size, and these could be blocked on the surface of the membrane or inside it. In this case, the dispersion of nanomaterials inside the polymeric matrix will enhance its performance for filtration by increasing the water flux or antifouling properties [104,105,106].

In addition to the above-described mechanisms, if the dispersed nanomaterials possess photocatalytic properties, contaminants adsorbed on the membrane surface or inside, in contact with them, could be degraded under irradiation, and the photodegraded by-products could be blocked by the membrane itself [29,30,107]. With respect to photocatalysis processes using semiconductor powders, this is an advantage since, in this case, toxic by-products are not released in water. Polymers could also positively affect the light absorption of the semiconductor itself, for example, extending it in the visible region of the spectrum or enhancing its photocatalytic performances [28,29,30,31].

The last but not less important advantage of polymeric nanocomposites is the possibility to block nanoparticles inside a matrix, avoiding their dispersion in the environment, having a material that can be easily removed at the end of the process, re-generated and re-used [27,28,29,108].

### 3.2. Desalination

The Nexar^TM^ polymeric architecture was investigated as a desalination membrane material: the transport properties, salt and water permeability, water uptake, and salt diffusion were studied and appropriately tuned by varying the degree of sulfonation, the block molecular weights, and film casting technique. By increasing the degree of sulfonation, water uptake and water permeability increased, resulting in a plasticization of the polymer and an increase in the water diffusion coefficient. Equal values of IEC and the size of the hydrophobic end block reduced water uptake. The sodium chloride salt permeability of the sulfonated pentablock copolymer materials increased with the salt concentration as an effect of the Donnan exclusion potential: at low salt concentration, its permeability was reduced by low chloride adsorption. When the salt concentration increased, the ionic strength was enough to overcome the Donnan exclusion effect, resulting in increased permeability [95]. Salt permeability is linearly dependent on high water content, i.e., high sulfonation degree. Anyway, the larger hydrophobic blocks restricted swelling and resulted in a material with increased selectivity. The transport properties of the material depend on the morphology and, therefore, on the casting method, as explained in the previous paragraph. This aspect is still under investigation. Geise investigated the sodium chloride diffusion properties in a steady state of sulfonated polymers with those of an uncharged hydrogel [96]. Compared with uncharged polymers, the salt diffusion coefficients of sulfonated polymers increased markedly as salt concentration increased. Further study is required to understand such trends fully.

A recently investigated simple and low-cost method for desalination is membrane capacitive deionization (MCDI), involving reversible electrosorption using high surface area porous electrodes paired with ion-exchange membranes [109]. The performance of an MCDI module depends strongly on the permselectivity and salt permeability of the membrane. These two parameters negatively affect each other. Indeed, permselectivity describes the preferential transport of cations over anions, or vice-versa, and permeability describes the rate of transport across the membrane. In order to obtain a good trade-off between these two properties, Nexar^TM^ was used in this field as a cation-exchange coating for MCDI electrodes because of its high density of sulfonic groups on a hydrophobic backbone [109]. Indeed, thanks to its molecular design, it is chemically, thermally, and mechanically stable, but the sulfonic groups confer it with the capacity for high water uptake to minimize ionic resistance while at the same time maintaining a high charge density of fixed charged groups to achieve a high permselectivity. This characteristic was investigated for Nexar^TM^ membranes coating electrodes in MCDI, confirming that Nexar^TM^ is an effective solution-processible ion-exchange layer for MCDI with tunable morphology, water uptake, and performance by varying casting conditions.

### 3.3. Pervaporation

Nexar^TM^ polymer was also used to prepare membranes for pervaporation processes. A pervaporation membrane is used for the separation process, which involves the partial vaporization of a liquid mixture through a dense membrane while the downstream side of the membrane is kept under vacuum. Zuo tested novel composite membranes obtained by dip-coating Nexar^TM^ on poly(ether imide) hollow fibers for the pervaporation–dehydration of C2–C4 alcohols [110]. The as-prepared membranes showed impressive separation performance: a higher IEC value induced enhanced hydrophilicity and stretched chain conformation, resulting in higher flux and lower separation factor. Moreover, the composition and structure of microdomains in the cast films depend on the solvent used in the preparation, which affects separation factors and fluxes.

Shi et al. used Nexar^TM^ copolymer as the polyanion on a hydrolyzed polyacrylonitrile (PAN) hollow fiber substrate in a new self-assembled polyelectrolyte multilayer membrane for ethanol dehydration with better hydrophilicity and water transport properties [111]. Nexar^TM^ allowed for a reduction in the required number of bi-layers while achieving a good separation performance. The newly developed PEMM showed a flux of 1160 g/m^2^ h and a separation factor of 127 at 50 °C for ethanol dehydration with just one bi-layer of polyethyleneimine (PEI) polycation and Nexar^TM^ polyanion.

Thomas also used Nexar^TM^ to prepare pervaporation membranes with desalination performances, such as excellent permeance and high salt removal, which are superior to commercial pervaporation membranes and equivalent to commercial membrane distillation membranes [112]. The pervaporation desalination performance was found to be only in part dependent on the polymer degree of sulfonation and casting solvent polarity, although these properties largely affect the membranes’ water uptake.

Recently, the same author developed a system for the management of brine for the International Space Station (ISS) wastewaters [113]: in this system, Nexar^TM^ was used as pervaporation membranes being previously coated by zwitterions (polymeric molecules with covalently tethered positive and negative ions). This modification enhanced the roughness and relative hydrophilicity of the membrane surface and reduced water passage as a consequence of higher thickness (after coating). The as-prepared membranes showed the potential to enhance the lifetime of the system and yield high recoveries over time.

### 3.4. Nexar^TM^ Films for Adsorption Process

Heavy metal and dye pollution has become a major problem in numerous countries, and the situation is expected to be further aggravated in the near future by rapid population growth and economic development. Facing stringent regulations on wastewater discharge containing heavy metal ions and organic dyes, various industries are demanding more efficient and effective treatment methods.

Adsorption is the transfer and accumulation of contaminants to a different phase without degradation [114,115,116]. When weak chemical interactions are involved between the adsorbate and the adsorbent, physisorption occurs. In contrast, these are chemically linked in chemisorption. Consequently, the last one is specific and irreversible, i.e., the chemical and electronic properties of the adsorbent are changed. The efficiency of an adsorption process is limited by the surface area and porosity of the adsorbent, and selectivity is lacking. Furthermore, material regeneration is usually expensive and results in the loss of the adsorbent. However, the development of new membrane materials is constantly required for the advancement of this technology.

#### 3.4.1. Heavy Metals Adsorption

Heavy metals are toxic and non-biodegradable, and they could bioaccumulate in the environment, posing serious risks to human health. Various treatment technologies such as ion exchange, adsorption, and membrane filtration have been adopted to achieve effective removal of heavy metals and recycling of wastewater [108,117]. In particular, filtration and/or adsorption by polymer films were extensively studied since these materials could provide high removal efficiency, easy operation, and fabrication at a low cost.

The use of Nexar as a selective layer for the development of a composite nanofiltration (NF) membrane for the removal of heavy metal ions was reported for the first time in [117]. The resultant NF membrane had a mean effective pore diameter of 0.50 nm, a molecular weight cutoff of 255 Da, and a reasonably high pure water permeability of 2.4 LMH/bar. Within these characteristics, the obtained membrane effectively removed both cationic heavy metals such as Pb^2+^, Cd^2+^, Zn^2+^, and Ni^2+^ and anions such as HAsO_4_^2−^ and HCrO_4_^−^ with removal efficiencies higher than 90%. The promising preliminary results achieved in this study provide a useful platform for the development of new NF membranes for heavy metal removal. Nexar^TM^ free-standing nanocomposite membranes were prepared for the removal of organic dyes and heavy metals by adsorption and/or photocatalysis [29,108].

Nexar^TM^ films (named s-PBCs) were prepared by the solvent casting method, redispersing the commercial polymeric solution commercialized by Kraton LLC [118] in polar solvent, i.e., dimethylformamide after the evaporation of commercial solvents. The polar solvent was chosen to increase the size of hydrophilic domains and induce ionic channels inside the film; this structure is preferred for water purification applications, i.e., adsorption or filtration processes [29]. The commercial Nexar solution was provided courtesy of Kraton Polymers LLC, and it is formed by a 10–12 wt% poly-(tBS-HI-sS:S-HI-tBS) polymer in a cyclohexane/heptane mixed solvent. The IEC value of this polymer is 2.0 meq/g, corresponding to a sulfonation degree of 52 mol%. The molecular weight is 112,500 g/mol, and the volume fraction of (tBS-[sS:S]-HI) is 0.300-[0.226:0.208]-0.266 [93].

In order to confer to this polymer higher adsorption properties and thermal/mechanical stability, hybrid Nexar nanocomposite membranes (sPBC-GO) were prepared by the solvent casting method, and adding graphene oxide (GO) flakes to the polymer solution [29,108]. GO is formed by graphene layers, including oxygen functionalities such as hydroxyl, epoxide, carbonyl, and carboxyl groups that increase the number of active sites for selective contaminants adsorption. The mixture was stirred until it was homogeneous and dense enough for casting on a Petri dish. After solvent evaporation, the film was removed by dipping in deionized water. The membranes were soaked and washed in deionized water (Millipore Advantage A10) at room temperature in order to remove eventual impurities, such as residual acids until the soaking solution stabilized at neutral pH. The as-prepared films were tested for the adsorption of heavy metals in water, such as Co^2+^, Ni^2+^, Pb^2+^, and Cr^3+^ ions.

**Figure 7:**
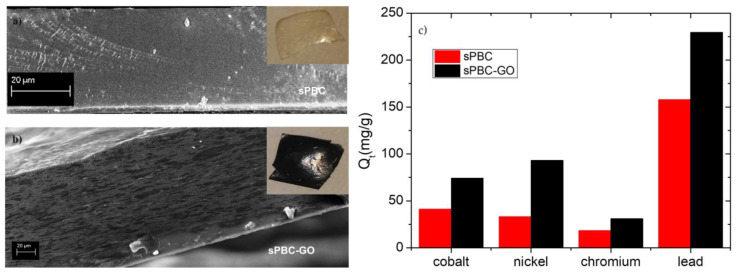
Cross SEM images and photos (insets) of a s-PBC membrane (**a**) and sPBC-GO composite membrane (**b**), respectively. In (**c**), the amount of adsorbed ions (mg) per gram of sPBC or sPBC-GO membrane, respectively, is reported. Images are reported from [108].

A very low amount of GO further improved the good adsorption abilities of the polymeric membrane without the release of contaminants into the environment [108]. Figure 7 reports the photos and scanning electron microscopy (SEM) images of prepared films with the amounts of ions adsorbed (as mg for gram of membrane).

As shown in Figure 7a, the s-PBC membrane is quite smooth and homogeneous; the presence of small irregular vertical lines in the SEM cross-section is due to the breaking of the membrane intentionally produced for cross-section analysis. In the case of s-PBC-GO (Figure 7b), the filler is dispersed throughout the entire volume of the polymeric matrix, showing a spongy structure with graphitic planes well visible. Furthermore, the color of the polymeric film turned from yellow to black after GO addition. The observed increased porosity of sPBC-GO with respect to sPBC itself, within GO hydrophilic character, resulted in an increase in the membrane’s water uptake values from 201% to 308% for s-PBC and s-PBC-GO, respectively. This aspect is fundamental in the application of these membranes in adsorption processes for water purification. Furthermore, the structure of s-PBC and s-PBC-GO films was fully characterized by XRD and SAXS measurements since the stability of these films and their adsorption properties are dependent on their structures [29,108]. These analyses confirmed that the GO flakes were completely dispersed inside the s-PBC matrix, and the films had a lamellar structure with a long period of the lamellar superstructure that increased from 40.8 nm to 51 nm. In other words, GO flakes occupy the space between lamellae and most probably in the transition layer between ion-rich and nonpolar lamellae. Furthermore, the good GO dispersion inside the sPBC polymer increased the mechanical and thermal stability of the composite: the storage modulus of the composite increased by around 30%, resulting in higher elastic properties without affecting polymer T_g_. The good dispersion of GO flakes resulted in an increase in the adsorption sites for heavy metals, as confirmed by the graph of mg of adsorbed ion for gram of membrane (Q_t_) values reported in Figure 7c. The s-PBC membrane itself was able to remove heavy metals by interacting with sulfonilic functionalities. The removal efficiency then follows the order Pb^2+^ > Co^2+^ > Ni^2+^ > Cr^3+^. Differences in the removal affinity and efficiency were observed after GO dispersion, in this case, following the order of Pb^2+^ > Ni^2+^ > Co^2+^ > Cr^3+^. Indeed, for Co^2+^, Cr^3+^, Pb^2+^, and Ni^2+^, the Q_t_ value increased by 1.8, 1.7, 1.5, and 2.8 times, with respect to those measured for the raw polymer [108].

In other words, higher Q_t_ values but also higher kinetic rate constants according to the diffusion model were observed for composites with GO because of a higher density of adsorption sites (sulfonic and oxygen functionalities) available for the ions and the introduction of morphological changes such as high porosity, high roughness and longer lamellar distances.

A direct comparison of adsorption abilities for different polymeric materials is not easy since these depend not only on the molecular structure of the polymers as the number of active sites for the adsorption, the morphology, and structure of the film, but also on the experimental conditions such as the weight ratio between adsorbent and metal ions to be removed, the pH of the solution, temperature, the nature of the counter ion. The above-reported results showed that using a small amount of GO dispersed in s-PBC (0.003% wt of GO with respect to s-PBC), a Q_t_ value of 74.2 mg/g for Co^2+^ removal was obtained, compared, for example, with the value of 68.2 mg/g for Co(II) removal on graphene oxide nanosheets reported by [119]. For Ni^2+^ ions removal, s-PBC/GO showed a removal efficiency of 93 mg/g, which is comparable with a value of 114.4 mg/g obtained by using a magnetically recoverable graphene/Fe_3_O_4_ composite [120].

The values reported above for Pb^2+^ ions removal (i.e., Q_t_ = 229.4 mg/g) were higher than most of the values reported in the literature. To provide some comparisons, the adsorption capacity for Pb^2+^ was estimated to be 76.94 mg/g for magnetic chitosan grafted with GO sheets [121], 100 mg/g for MnFe_2_O_4_^−^ graphene composite [122], and 166.66 mg/g using carbon nanofiber grown on powered activated carbon [123].

Concerning polymeric materials, the Nafion adsorption efficiency of Pb^2+^ ions was estimated to be lower by about three times than for s-PBC in terms of Q_t_ [124]. This can be ascribed to the higher number of active sites (i.e., sulfonic groups) present in Nexar than in Nafion chains, underlying their high affinity with Pb^2+^ ions. A deeper discussion on the direct comparison of these two polymers for dye removal is reported in the following paragraphs, and experiments were performed under the same conditions.

#### 3.4.2. Dyes Adsorption

**Figure 8:**
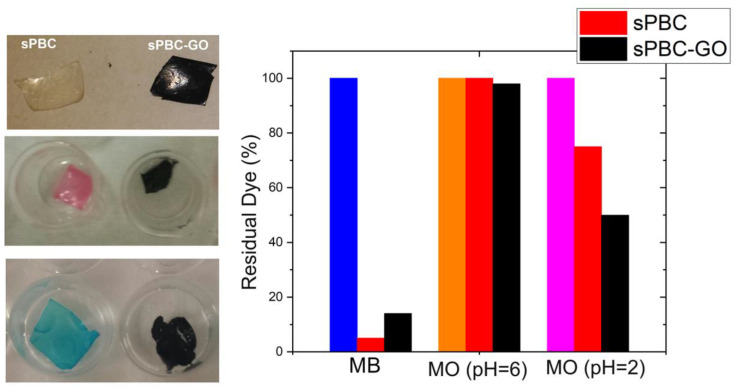
Photos (on the left) of membranes before and after the MO and MB adsorption experiments and the residual amount (%) of dyes after membrane adsorption (on the right). The adsorption experiment of MO was conducted at both neutral and acidic pH values. Blue, orange and pink bars refer, respectively, to the MB, MO at pH = 6 and MO at pH = 2 solutions before being in contact with the membranes. Reproduced from [29] with permission from the Royal Society of Chemistry.

More than 100 highly toxic and potentially carcinogenic dyes are still available on the market, and around 15–50% of azo-type textile dyes are released into wastewater during the dyeing process [125,126]. If we consider that this water is commonly used in developing countries for the purpose of irrigation in agriculture [127,128], it is easy to understand the elevate risk of bioaccumulation and causing many diseases in humans, such as dermatitis, disorders of the central nervous system [129], or to the inactivation of enzymatic activities themselves by the substitution of enzymatic cofactors [130].

s-PBC membranes were also tested by [29,31] for the removal of anionic and cationic azo dyes in water by adsorption and photocatalysis (reported in the next paragraph). The s-PBC and s-PBC-GO membranes were immersed in the dark in an aqueous solution containing a cationic dye, methyl orange (MO), and an anionic one, methylene blue (MB), respectively. Figure 8 reports the photos of membranes after the adsorption experiments and the residual percentage (%) of dyes after membrane adsorption.

As shown in Figure 8, the s-PBC membrane changes its color to blue or red due to the interaction with MB or MO, while in the case of s-PBC-GO, no evident color change is observed since its initial color is black. For MO, the membranes became red, evidencing their acidity since MO is orange at neutral pH and became red at pH < 3. Filice et al. reported in [29] that, unlike the use of powders directly dispersed in water, MB is removed immediately by the membranes and without the formation of any precipitates or flocculates. The good removal efficiencies were explained considering the electrostatic attractions between the MB positive superficial charge and the negative charge of the sulfonilic groups present on the membranes: all the materials were able to remove more than 90% of the initial MB concentration.

In contrast, the adsorption of anionic MO dyes is hindered because of electrostatic repulsion. As shown in Figure 8 on the right, s-PBC and its composites were not able to adsorb the dye at neutral pH, while at the pH value of the dye solution down to 2, s-PBC was able to adsorb more than 20% of the initial MO concentration after three hours. At an acidic pH, MO has a positive charge derived from its protonation, and thus, it could interact with negative sites on membranes. GO enhanced the MO adsorption that passes from 20% for the filler-free polymer to 50% for the composite.

These results are the basis for the use of these membranes in photocatalytic processes for azo dye degradation, as reported in the following paragraph.

### 3.5. Nexar^TM^ Photocatalytic Nanocomposites for Dyes Degradation

After being removed by water, the contaminants, such as microorganisms and organic molecules, could be converted into fewer toxic products through chemical/biological oxidation and advanced oxidation processes [131]. The complete mineralization of organic and inorganic substances into CO_2_, water, and mineral acids could occur by reaction with hydroxyl radicals (·OH). The processes based on those reactions are known as advanced oxidation processes (AOPs) and refer to a set of chemical treatment procedures performed at normal pressure and temperature [132]. Hydroxyl radicals are produced with the help of one or more primary oxidants (e.g., ozone, hydrogen peroxide, and oxygen) and/or energy sources (e.g., ultraviolet light) or catalysts (e.g., titanium dioxide). Although these methods are highly efficient and do not involve the formation of toxic by-products, AOPs still have not been put into commercial use on a large scale (especially in developing countries), mostly because of the relatively high costs and low selectivity.

In this regard, photocatalysis has been recognized for its high capability to mineralize organic compounds in an efficient, green, and cheaper manner [133,134]. During a photocatalytic process, a semiconductor such as a metal oxide is irradiated with light equal to or higher than its energy gap, generating electron-hole pairs. In addition to their recombination, the electrons and holes are reducing and oxidizing agents, respectively, that could generate hydroxyl radicals in the presence of water. These radicals are responsible for contaminant degradation. Usually, UV light is used for this process, but many recent studies are devoted to the appropriate engineering of semiconductors to be active under visible light in order to reduce the costs [135].

**Figure 9:**
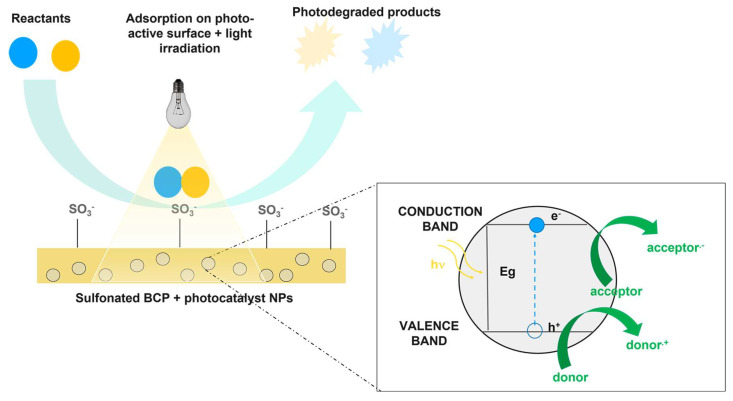
Scheme of photodegradation of water contaminants by Nexar nanocomposites under light irradiation. A scheme of the photocatalytic process is shown in the box.

Starting from previous results showing that Nexar film interacts through sulfonic groups with MO and MB dye molecules, these could also be used to synthesize photocatalytic membranes by dispersing inside the polymer photocatalytic nanoparticles. Indeed, the first step to achieving photocatalytic performance is to degrade the contaminant in direct contact with the active material. Furthermore, the immobilization of nanostructured photocatalysts in a polymer matrix allows for the avoidance of particle dispersion. Nexar nanocomposite membranes have been tested by [29,31] for the photodegradation of organic dyes (i.e., MO and MB) under UV or visible light. In this case, the semiconductor nanoparticles were dispersed in the polymeric matrix, and the nanocomposite film was immersed in a dye solution under irradiation. The dyes were degraded according to the mechanism reported in Figure 9.

Contaminant molecules were adsorbed on the polymeric surface through the electrostatic interaction with the sulfonilic groups on the polymer backbone, which also favored the interaction with the photocatalyst inside the polymeric matrix. The photocatalyst degraded the adsorbed molecules by the generation of electron/hole pairs as a consequence of light adsorption (see the box in Figure 9). The by-products of degradation could be released in solution and could remain adsorbed on the membrane surface.

For the first time, photocatalytic s-PBC membranes were prepared by dispersing active UV metal oxides inside it, i.e., titanium dioxide (TiO_2_) and bismuth oxide (Bi_2_O_3_), and their photodegradation activity was tested for the removal of both MB and MO [29,31]. Their removal efficiencies were compared with those of s-PBC and s-PBC-GO membranes used for the same photodegradation process. Since all s-PBC membranes showed a removal efficiency higher than 90% in the dark, no particular effect on the total removed amount of MB molecules was observed under irradiation. The main differences between adsorption and photocatalysis were observed, taking into account the kinetic and removal efficiencies of MB monomer, dimer, and aggregates, respectively, for each material and process [29]. Indeed, by deconvolution of MB absorbance spectra in the main three peaks related to monomer, dimer, and higher aggregates, the authors affirmed that the kinetic of monomer and aggregate formation depends on the type of process and the specific material. All the membranes were able to remove monomers and dimers with time, independently of the specific process. Higher aggregates were formed in the first minutes of contact with the membranes, in particular for s-PBC-GO, and the higher efficiency in their removal/degradation was reported under irradiation.

**Figure 10:**
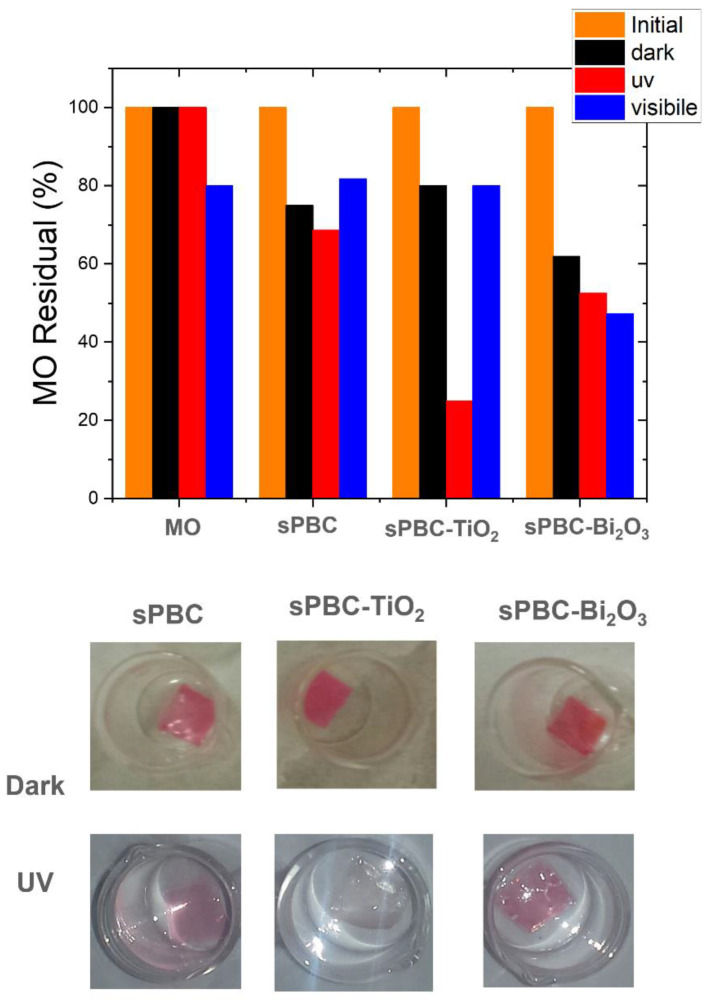
Percentages of MO removal by different s-PBC nanocomposite films under UV or visible light irradiation (on the top). Images of membranes after adsorption or photocatalytic processes (on the bottom). Experiments were conducted at pH = 2. Partially reproduced from [29] with permission from the Royal Society of Chemistry and from [31] with permission from Elsevier.

In contrast, with respect to MB, the MO dye is a perfect candidate to be used as a model compound to study the photocatalytic activity of Nexar membranes since its adsorption is low or totally hindered, as previously reported. Consequently, the observed MO degradation under irradiation is due to photocatalysis and not to adsorption. Furthermore, MO degradation could be easily followed by acquiring UV-Visible absorbance spectra of dye solutions where the membranes were immersed under irradiation. Figure 10 reports the percentages of MO removal by different s-PBC nanocomposite films under UV or visible light irradiation. These values are compared with the removal percentages obtained in the dark. Photos of membranes after adsorption in dark and photocatalytic processes under irradiation are reported, respectively.

As observed in Figure 10, in acidic conditions, s-PBC membranes were able to adsorb protonated MO molecules, and MO photodegradation occurred under UV or visible light. S-PBC and sPBC-TiO_2_ were able to adsorb more than 20% of the initial MO concentration after three hours. Under irradiation, the removal efficiencies increased for all materials, and the best performance (about 75%) was reported in the composite with TiO_2_. In particular, all membranes became red after adsorption, while they remained white after the photocatalytic process even if MO removal occurred (see Figure 10 on the bottom) [29,31]. These confirmed that in the dark, the membranes were able to adsorb MO molecules while under irradiation; their removal occurred by photodegradation. This was also confirmed by the authors observing the MO solutions absorbance spectra after irradiation processes in the presence of polymeric films: a peak below 300 nm was observed, suggesting the formation of by-products generated by photocatalytic degradation.

In Figure 10, the use of the as-prepared polymeric matrix for visible light photocatalytic application is reported. In this case, only the s-PBC-Bi_2_O_3_ composite reported a good removal performance of up to 50% for MO degradation (see Figure 10). According to [31], this activity was due to the formation of a mixture of Bi-Bi_2_O_3_ as an effect of the reduction of Bi_2_O_3_ during the membrane preparation, and this enhanced the photocatalytic activity under visible light irradiation.

The s-PBC-GO membrane was tested for photocatalytic degradation under UV-Vis irradiation: by comparing the results obtained for s-PBC-GO and s-PBC TiO_2_, the authors stated that the two films had the same removal efficiencies under irradiation, but in the case of s-PBC-GO, no toxic by-products due to MO degradation were observed [29]. In this study, the possible use of Nexar^TM^ as a matrix to prepare polymeric nanocomposites to be used in water was shown within the advantage of using GO to increase the removal performances without forming toxic by-products.

**Figure 11:**
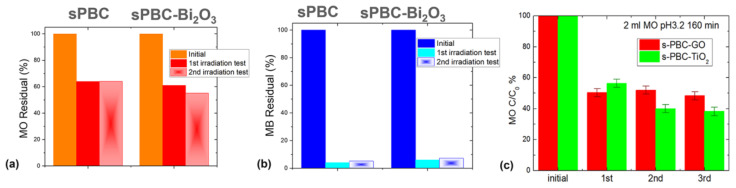
Consecutive cycles of adsorption and/or photocatalytic processes for MO (**a**,**c**) and MB (**b**) removal by different polymeric nanocomposites after their regeneration. Partially reproduced from [29] with permission from the Royal Society of Chemistry and from [31] with permission from Elsevier.

Furthermore, all prepared polymeric films could be easily washed in water and used again in adsorption or photocatalytic processes [29,31]. Figure 11 reports consecutive cycles of adsorption and/or photocatalytic processes for MO and MB removal by different polymeric nanocomposites after their regeneration. The results show that similar removal performances can be achieved for regenerated membranes.

**Figure 12:**
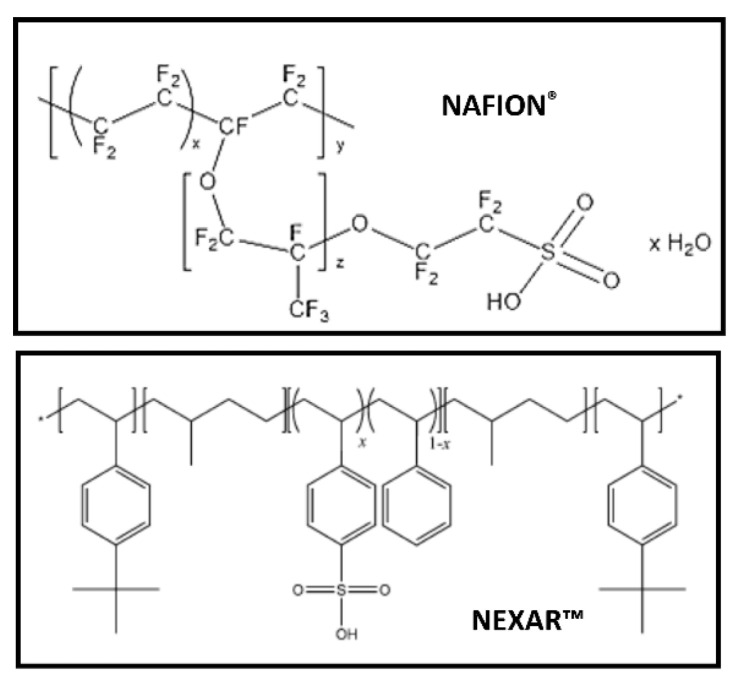
Scheme of the Nafion^®^ and Nexar^TM^ polymer, respectively. Reproduced from [93].

Results obtained in the application of Nexar^TM^ polymeric nanocomposites in water purification were compared with results obtained using another well-known sulfonated polymer, i.e., Nafion, in order to show that not only Nexar^TM^ is cheaper than Nafion but higher results could be achieved using it. The structure of Nafion is reported in comparison with the one of Nexar in Figure 12.

Table 2 reports a comparison between the performances of Nexar and Nafion nanocomposites for MB and MO removal by adsorption or filtration.

First of all, Nexar^TM^ has a higher acidity and hydrophilicity than Nafion, as confirmed by the water uptake values (i.e., 24% for Nafion and 201% for Nexar). This is ascribed to the higher density of sulfonated groups for Nexar^TM^ with respect to Nafion. With regard to MB removal, both Nafion and Nexar^TM^ reported high removal efficiencies: in both cases, higher removal efficiencies are favored by electrostatic attraction between sulfonic groups and positively charged dye molecules. Consequently, Nexar membranes show higher efficiencies since they have a higher sulfonic group density. The only difference is that no MB aggregation occurred using Nafion, and this is explained by considering a different density and structural distribution of active adsorption sites in Nafion with respect to Nexar^TM^. The higher density of sulfonic groups in Nexar^TM^ membranes, i.e., negative charge, is responsible for the fact that Nexar is unable to adsorb MO at neutral pH but only under acidic conditions, i.e., when MO is protonated. In contrast, concerning MO removal, Nafion could adsorb a moderate amount of MO (45% in the same experimental conditions) [28]. Furthermore, the degradation rate for MO increased under irradiation for both Nexar and Nafion layers. The dispersion of active nanoparticles such as titanium dioxide and graphene oxide flakes highly enhanced the photodegradation ability of the nanocomposites, with the best results reported for Nexar with respect to Nafion.

To sum up, the Nexar-based materials, showing comparable activity with Nafion nanocomposites, are proposed as a cheaper and safer alternative to Nafion, in particular considering their preparation methodology. This is true not only for water remediation purposes but also for their use in energy applications as proton exchange membranes, as reported below in the last section of this Review. Nexar^TM^ membranes were shown to be very effective and selective in removing cationic contaminants. The addition of nanoparticles could confer higher adsorption efficiencies or new functions as photocatalytic properties to this polymer while still maintaining unaltering polymeric matrix features. Furthermore, both Nexar membranes could be easily washed and used again.

## 4. Nexar as Coating Layer of Commercial Filters

Polymeric membranes have wide applications in filtration technology for air and water purification in different industrial fields. Filtration is the process by which some molecules are blocked by a filter depending on their size or by other kinds of physical or chemical interactions while other molecules are allowed to pass through. Filtration is effective in removing impurities, contaminants, and solids from liquids and air, and it is a green process. The main disadvantages are the high costs due to the high pressure and energy required and the maintenance and replacement of the filters. One approach to building low-cost but highly efficient filters is the coating of commercial low-cost filters by selective and active coating layers made up of other polymers with or without the addition of nanomaterials.

Nexar has shown high hydrophilicity, antimicrobial properties, and a high density of sulfonic groups that confer the polymer selective and high-performance adsorption properties. All these characteristics make it a good candidate as an active coating layer for commercial filters in order to reduce the biofouling of hydrophobic polymer and increase its selectivity and performance in the filtration process for anionic/cationic species.

### 4.1. Antimicrobial and Antibiofouling Properties of Nexar

Recently, the antimicrobial properties of Nexar^TM^ polymer have been investigated. Of particular concern for global healthcare is antimicrobial resistance: many pathogens develop resistance to conventional medical treatment, which is seriously dangerous for human health and highly affects the costs of medical services. Continuous efforts to develop efficient self-disinfecting materials are necessary [136]. Recently, the self-disinfecting antimicrobial property of Nexar^TM^ without the need for additives was reported in [34]. Due to its high acidity, this sulfonated copolymer was capable of inactivating Gram-positive and Gram-negative bacteria and was also highly effective against spore-forming bacteria. The antimicrobic activity of this polymer can be fully rejuvenated to its maximum performance level by relatively short immersion in acidic solutions.

Furthermore, it was shown that polymer morphology, which can be templated by the casting solvent or altered upon exposure to solvent vapor or liquid water, affects inactivation efficacy. Anyway, the nature of the mechanisms involved in the antimicrobial activity was not clear [137].

**Figure 13:**
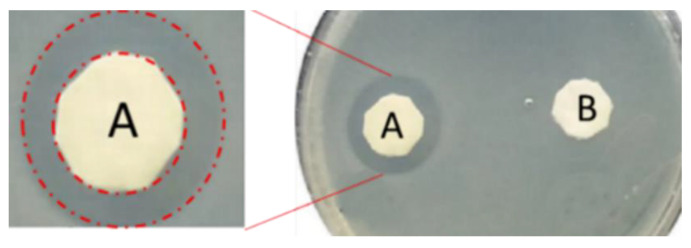
Modified Zone of Inhibition Test of *P. aeruginosa* after 24 h incubation with coated (A) and uncoated (B) filters in the presence of water. Partially reproduced from [32].

Sciuto et al. proposed the use of Nexar^TM^ (s-PBC) as an innovative multifunctional coating for improving the performance of commercial polypropylene filters [32,33,138]. The authors tested the survival of *P. aeruginosa* after its exposure to uncoated and coated filters. The bactericidal activity of s-PBC coating was deeply investigated by a modified Zone of Inhibition Test in which s-PBC@PP and reference coupons were directly faced on top of a *P. aeruginosa* plate and incubated for 24 h in dry and wet conditions. Figure 13 reports the Modified Zone of Inhibition Test of *P. aeruginosa* after 24 h incubation with coated and uncoated PP filters in the presence of water.

In the last case, the s-PBC coating showed an inhibition effect towards the *P. aeruginosa* proliferation, as evidenced by a clear zone appearing all around the coated filter. The observed halo could be ascribed to the acidification of a small volume of water in contact with the filter surface induced by the presence of sulfonic groups in the coating layer. In addition to the as-shown antimicrobial activity, the covering of PP with s-PBC results in a more hydrophilic, acid, negatively charged, and smoother surface. Thanks to these properties, the adhesion and proliferation of Pseudomonas were negated, and an evident antibiofouling activity was observed.

The principal problem of membrane filtration affecting their lifetime, performances, and the costs of the process is membrane fouling [35]. Fouling is the accumulation of solid particulates, micro-biological organisms, and dissolved organic components/colloids on the membrane surface that create a compact film or could penetrate inside the membrane, causing pores occlusion, i.e., higher pressure for the filtration is required and the filter lifetime is reduced. Building antifouling membranes is necessary to avoid the adhesion of micro-biological and organic compounds on the filter surface and/or to kill bacteria, preventing them from forming a biofilm. For these purposes, hydrophilic surfaces with antimicrobial properties are needed. An alternative route to the use of hydrophilic films that have lower mechanical resistance than hydrophobic ones is the coating of commercial low-cost hydrophobic filters with hydrophilic surfaces.

**Figure 14:**
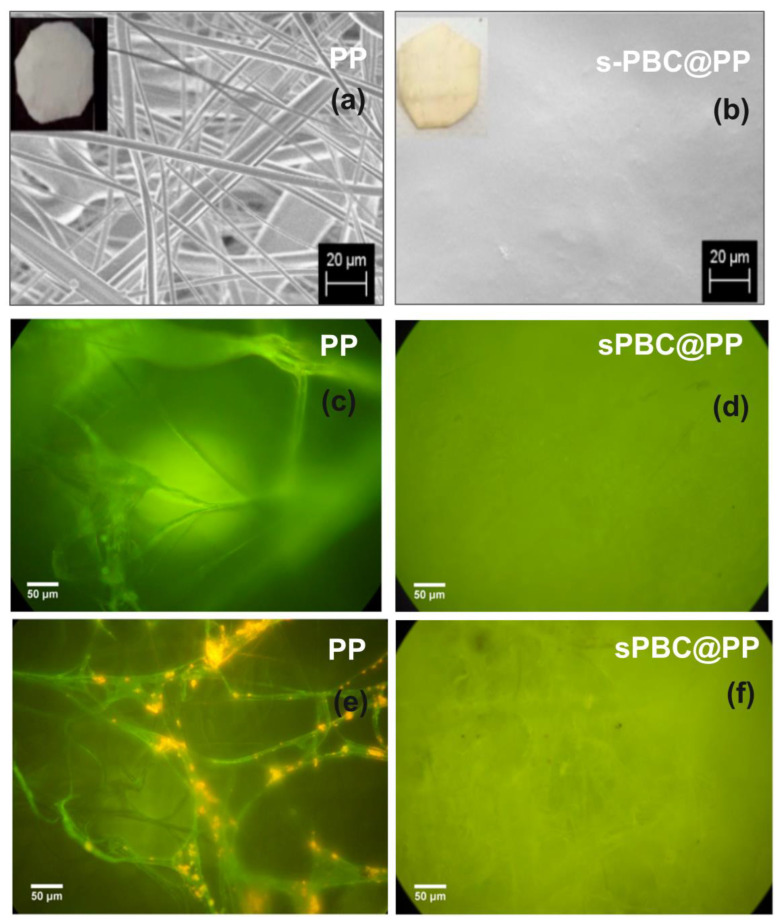
Photos and SEM images (**a**,**b**) of polypropylene (PP) (on the left) and sulfonated pentablock copolymer (s-PBC)@PP (on the right) coupons. Fluorescence optical microscopy images of reference PP and s-PBC@PP coupons before (**c**,**d**, respectively) and after (**e**,**f**, respectively) 20 days of incubation with *Pseudomonas aeruginosa* in water. Partially reproduced from [32].

Figure 14 reports the SEM images (a,b) and fluorescence microscopy photos (c–f) of initial and coated filters before (c,d) and after (e,f) exposition to *Pseudomonas aeruginosa* in water.

As evidenced in images b and d of Figure 14, s-PBC deposition completely covers the initial fibrous filter, resulting in a homogeneous and smooth surface able to reduce the bacteria’s chance of attachment on the filter surface. Indeed, once exposed for 20 days to the microbial suspensions, no traces of bacteria (yellow aggregates) were visible on the exposed surface when it was covered by Nexar (Figure 14f), unlike the uncoated filter (Figure 14e).

**Figure 15:**
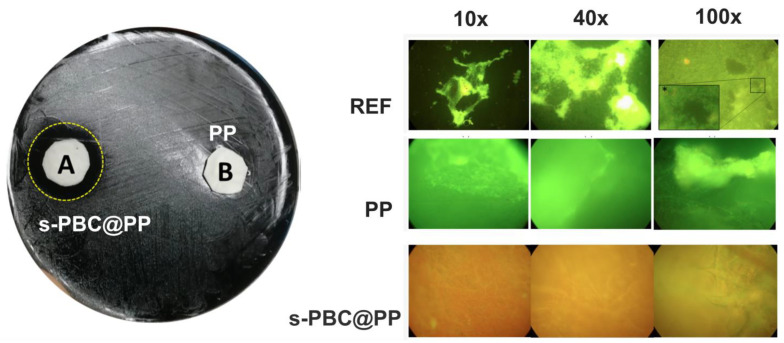
The modified Zone of Inhibition test (on the left) and biofilm formation tests (on the right) using coated and uncoated polypropylene (PP) coupons (named A and B, respectively) against *Legionella pneumophila* SG 2–16. A reference was used for biofilm formation tests. Partially reproduced from [33].

The same filters were tested for antimicrobial and antibacterial effects against another pathogen [33]. Figure 15 reports the modified Zone of Inhibition test and biofilm formation tests using the same material, polypropylene (PP) coupons, against *Legionella pneumophila* SG 2–16.

After 24 h, a clear zone appeared all around the coated filter, still confirming its antibacterial activity (left panel). Similarly, no biofilm was formed on coated filters (s-PBC@PP) thanks to the presence of sulfonated copolymer, while the biofilm was observed on the reference (REF) and uncoated filters (PP) (right panel). These images confirmed that the treated surface blocked any attachment attempts of the suspended cells.

The antimicrobial and anitbiofouling activity of Nexar coating could be ascribed to the presence of acid sulfonilic groups. Moreover, in [32], the authors deeply investigated this aspect to identify the antimicrobial mechanism by comparing the results obtained with neutralized Nexar-coated filters or by repeating the same experiment in large water volumes or solutions at a controlled neutral pH.

**Figure 16:**
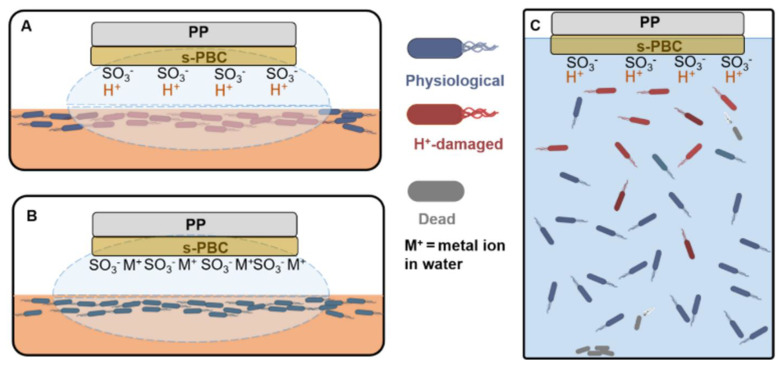
Schematic representation of *P. aeruginosa* death induced by s-PBC@PP and water: (**A**) acid s-PBC@PP in a small volume system; (**B**) neutralized s-PBC@PP in a small volume system; (**C**) acid s-PBC@PP in a large volume system. Physiological (blue), damaged (red), and dead (gray) bacteria are indicated with different colors. Partially reproduced from [32].

They also observed the antimicrobial activity of Nexar-coated PP filters when these were immersed in large water volumes or when their surfaces were neutralized. The authors explained the observed antimicrobial activity of Nexar coating according to the mechanism reported in Figure 16. In small water volume systems (0.2–0.5 mL) used for the Zone of Inhibition Test (see Figure 16A), the pH decreased to acidic values; hence, plated *P. aeruginosa* replication is inhibited within the water drop (red-colored bacteria in Figure 16A) producing the clear zones. In contrast, for the same water volume and in the presence of neutralized coated filters (Figure 16B), water pH does not change, and the bacteria survive and regularly proliferate (blue-colored bacteria in Figure 16B).

In large water volume systems (Figure 16C), such as those used for the biofilm formation assay (20 mL), the pH solution was fixed above the neutral value even if the coated filter was immersed. In this case, bacterial death was induced by the fact that the mobility of physiological *P. aeruginosa* (blue) in water was sufficient for them to approach the acidic surface of s-PBC@PP: near the surface, the released H+ ions interact with bacteria, inducing cell damage (red) and death (gray).

The results pointed out the possibility of using Nexar^TM^ as a coating layer with antifouling properties and antimicrobial activity.

### 4.2. Nexar Coated PP Filters for Heavy Metals and Dyes Filtration

The innovative Nexar membranes or coatings described above represent a promising solution for the simultaneous removal of different kinds of contaminants (organic and inorganic) and for avoiding biofilm formation. Having proved its inhibition effect on bacteria physiology, the same authors of [32,33] tested the smart s-PBC polymer as a potential coating for water filters. Their filtration abilities and antibiofouling properties were compared with commercial filters.

*L. pneumophila*-contaminated tap water samples were filtered through coated and uncoated commercial PP filters, and the bacteria found in filtered water, as well as the colony density present on filters after the filtration process, were compared for both filters [34]. The results showed a higher bacteria removal efficiency by the modified filter than the uncoated one as a consequence of the more compact layer formed by s-PBC, which reduces the mesh. No biofilm was formed on coated filters.

**Figure 17:**
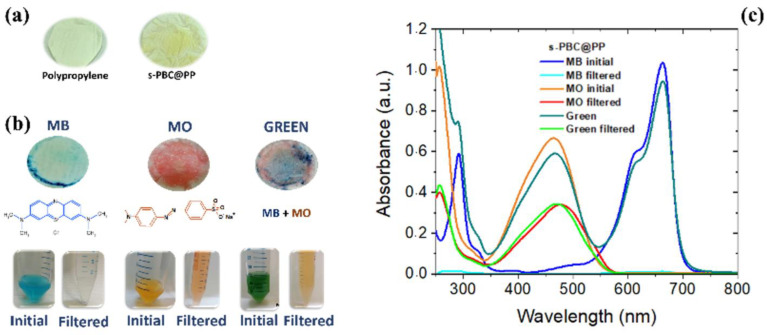
(**a**) Photos of PP and s-PBC coatedPP filters before the filtration of MB, MO, and mixed (Green) solutions, as well as (**b**) coated filters after filtration of MB and MO dye solutions. For each dye, the scheme of the dye and the images of initial and filtered solutions are reported. (**c**) UV-Visible spectra of dye solutions before and after filtration through a coated filter.

The same filters were tested for the removal of cationic dyes and heavy metals. Figure 17 reports the images of commercial and coated filters with a layer of Nexar before and after the filtration of cationic and anionic dye solutions and the relative UV-Visible absorbance spectra.

The commercial and coated filters were tested for the selective removal of cationic molecules by testing their filtering abilities for a cationic or anionic dye, MB or MO, respectively, in single or mixed dye solutions. Commercial polypropylene filters were ineffective in removing dyes. The coating enhanced the filtration abilities of commercial PP and conferred selectivity for the cationic dye. As evidenced by both images and UV-Visible spectra acquired on solutions before and after filtrations, MB was totally removed by filtration while MO was only partially blocked on the filter (i.e., 50%). The same results were observed for mixed dye solution (here named Green), confirming the selectivity of the coating layer towards cationic species.

**Figure 18:**
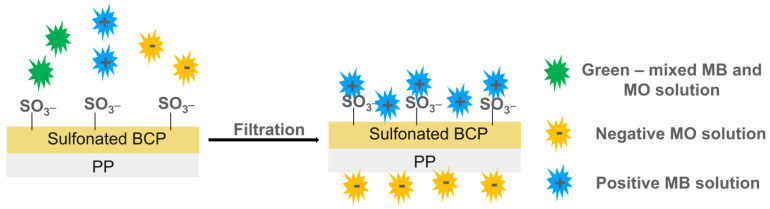
Scheme of the selective adsorption of positively charged molecules (MB) with respect to negative ones (MO) by Nexar coated PP filters during an adsorption/filtration test of mixed dyes solution (Green).

The adsorption/filtration mechanism results in the selection of positively charged molecules with respect to negative ones and a scheme for this mechanism is reported in Figure 18.

Positively charged contaminants are removed by direct interaction with the negative active sites on the polymeric backbone (i.e., sulfonilic groups), while the anionic species adsorption is hindered by electrostatic repulsion. In the filtration process, the anionic contaminants pass through the membranes, while in the adsorption process, these remain in the solution. In mixed dyes solution, the interaction with the Nexar surface induces the separation of MO and MB dyes by the selective adsorption of MB on polymeric active sites while the MO molecules pass through the filter.

In order to enhance the filtration abilities of coated PP filters, their surface was coated with a composite solution formed by Nexar polymer and graphene oxide in dimethyl formamide (DMF) to ensure good dispersion of GO flakes inside the polymeric layer. GO was added to enhance the surface area and the negative active sites for selective filtration/adsorption of contaminants. Coated filters with different weight percentages of GO were prepared and tested for the adsorption/filtration of iron ions.

**Figure 19:**
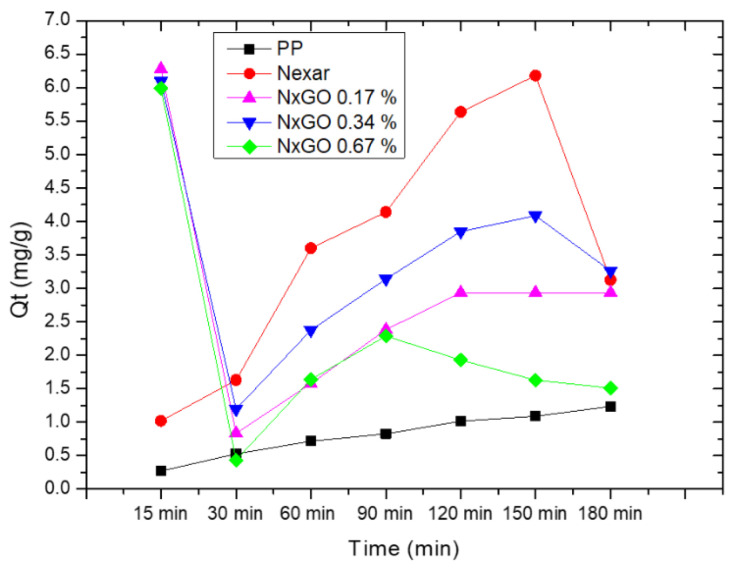
Q_t_ values of Fe^3+^ ions versus adsorption time for uncoated and coated filters immersed in FeCl_3_ solutions. The composite layer was investigated considering three different amounts of dispersed GO flakes.

Figure 19 reports the amount of adsorbed ions in mg for gram of coating layers (Q_t_) as a function of time, considering the adsorption process in the dark by immersing the uncoated and coated filters in FeCl_3_ solutions. The composite layer was investigated considering three different amounts of dispersed GO flakes.

As shown in the above figure, PP and modified PP filters were immersed in a FeCl_3_ solution (5 mL, 1.1 mM), and the Fe^3+^ adsorption process was followed over time by UV absorbance measurements. An equilibrium state was reached after 180 min. The Q_t_ values are larger for all coated filters than for uncoated ones, confirming that the coating layer increased the removal efficiency. By comparing the curves in Figure 19, different slopes can be observed, indicating different adsorption kinetics, i.e., different interactions between the ions and the active filter layers. In particular, the Q_t_ for the s-PBC@PP filter (red line) showed a linear increase up to a maximum value that was reached after 150 min. The curves for filters coated by the composite layers showed the maximum Q_t_ value at 15 min, then it sharply decreased and increased again until it reached equilibrium. This behavior is the same for all composite layers independently from the amount of dispersed GO flakes, and it was due to the saturation of the surface of the filters, resulting in the release of ions into the solution. Table 3 reports the Q_t_ values at 15 min and 180 min for all investigated filters during the adsorption process.

It is evident that the coating of the PP filter improved its performance in Fe^3+^ adsorption as an effect of the active sites (i.e., sulfonic groups of the polymer itself and charged negative moieties on GO flakes) present on the coating. Adsorption was greater in the presence of GO since the negative charge on the filter surface increased because of the presence of carboxyl and hydroxyl groups. The use of the s-PBC-GO composite coating allowed for obtaining higher retention of ions in just a short time (15 min), while the s-PBC coating exhibits the best performance at 150 min. After 15 min, the ions started to be released by the composite layers, and the final adsorption values were comparable with the ones obtained by the Nexar layer. The rapid release of ions once saturation is reached suggests that the ions are unable to permeate through the filter layers, so adsorption occurs only as a result of surface interactions. An increase in the amount of dispersed GO flakes did not improve the adsorption properties. The results showed that Nexar coating not only conferred the PP filter with a removal ability toward iron ions but also that the dispersion of a small amount of GO in the polymer is sufficient to increase up to six times the adsorption efficiency of Nexar and to reduce by one order of magnitude the time required to obtain the maximum adsorption.

In the next studies, Filice et al. investigated the role of the dispersing solvent of the coating layer passing from DMF to IPA in order to use a solvent that is greener and less aggressive for PP fibers [139,140]. The as-prepared filters were tested for the removal of heavy metals (Co^2+^ and Fe^3+^) in adsorption and filtration processes. The amount in mg of adsorbed ion per gram of coating layers (Q_t_) is reported in Table 4.

The negative charge and acidic character of polymeric layers conferred high removal efficiency to inactive PP filters for all investigated contaminants. The dispersion of GO flakes into the s-PBC polymeric layer had an evident effect only on the adsorption of Co^2+^ ions. This depends on the chemical affinity between GO and metal ions and on the nature of GO flakes dispersion in the polymeric matrix. Filice et al. investigated the mechanisms involved in the removal processes of metal ions by characterization of the filters before and after use using FT-IR and EDX analysis [139,140]. They observed that the removal processes occurred by interaction with sulfonic groups of the polymeric layer, and they evidenced that in the adsorption processes, due to longer interaction times with respect to filtration, the formation and release of new species in solution occurred.

### 4.3. UV Treatment of Nexar Coated PP Filters

In order to explore the possibility of further increasing the filtering efficiency of s-PBC coating, UV irradiation was performed [140]. UV irradiation has recently become popular as an easy and low-cost method to modify membrane surfaces for various industrial and biological applications [141,142,143]). For example, in industries, applications of UV may include disinfection of surfaces, curing, and activation of surfaces [142]. In particular, UV surface treatment increases the surface hydrophilicity: Quoc Toan Le reported that a fluorocarbon polymer became more hydrophilic after UV treatment as a consequence of the decrease in the fluorine content within the formation of carbonyl groups [144]. Similarly, different polymer surfaces were converted from hydrophobic to hydrophilic and vice versa by UV irradiation in a controlled atmosphere [145]. UV-induced modification can be divided into (i) UV-grafting using monomers with hydrophilic character and (ii) UV-induced changes using nanoparticles, for example, titanium dioxide that is active under UV irradiation and characterized by super-hydrophilicity [142]. In both cases, the final aim is to increase the wettability of the polymer surface.

Similarly, UV irradiation was used to increase further the wettability, antifouling, and filtering properties of commercial polypropylene filters coated with Nexar/Nexar-GO used for dyes and heavy metals removal [139,140]. The coating surface became more hydrophilic after UV treatment, as shown by the contact angle measurement and FT-IR spectra. This resulted in improving the removal ability of Co^2+^ ions and Fe^3+^ ions after just one single filtration step. In addition to this result, after UV irradiation, the surface showed the ability to interact with water contaminants, modifying them.

**Figure 20:**
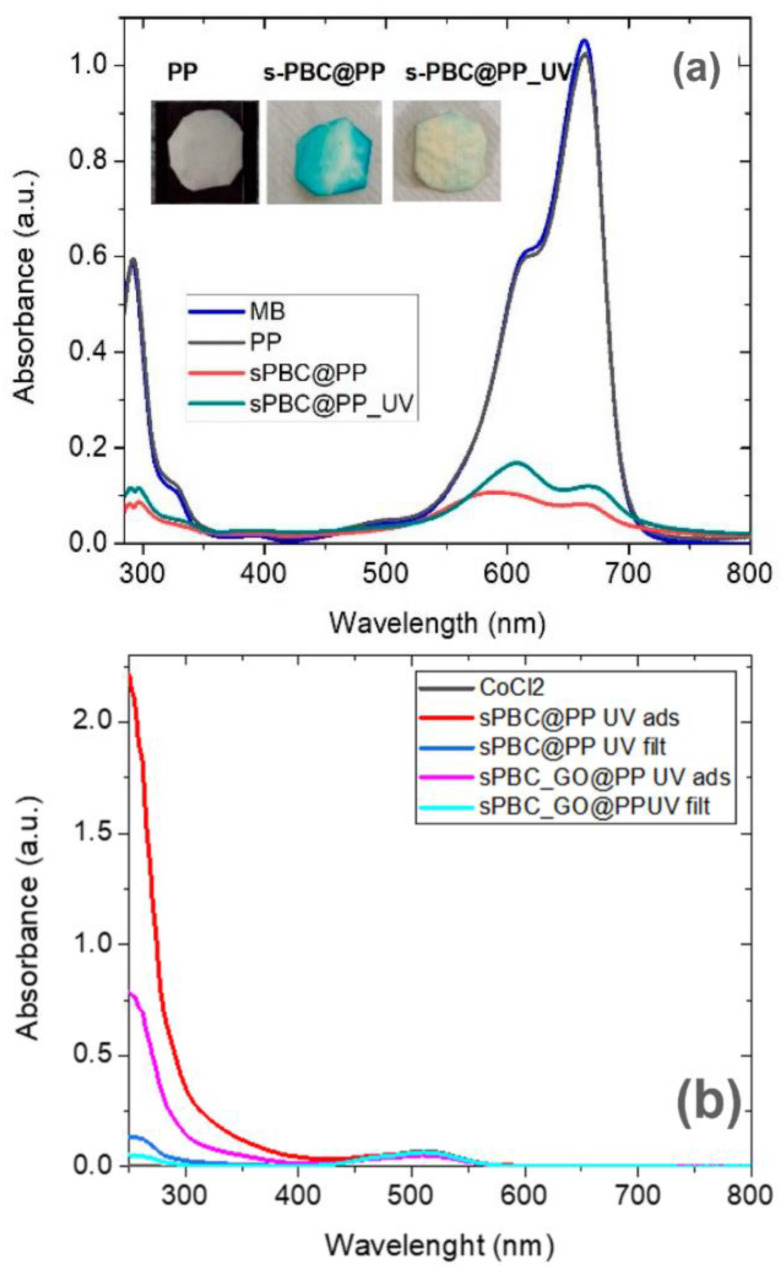
Images of commercial (PP) and coated filters untreated (s-PBC@PP) or UV-treated (s-PBC@PP_UV) after the removal of MB molecules by adsorption (**a**). UV-Visible spectra of cobalt solution where UV-treated and untreated filters coated with s-PBC/sPBCGO were immersed to remove Co ions (**b**). Reproduced [139,140].

Figure 20 reports the images of filters, UV-treated and not, after the removal of MB molecules and the UV-Visible spectra of cobalt solutions where the same filters were immersed to remove metal ions.

In the case of methylene blue removal, for example (see Figure 20a), the UV-irradiated filter removed the dye with the same efficiency as the untreated one, but its color did not turn blue after MB adsorption. This was explained as follows: the UV treatment of the coating enriched its surface with electrons that reduced MB molecules to the colorless form (i.e., leuco) directly into the solution or after surface adsorption [139].

When the UV-treated filters were used for the removal of Co^2+^ ions by adsorption or filtration, a new peak at 256 nm was observed as a consequence of the release of new species due to the interaction of cobalt ions with the treated coating surface (see Figure 20b). The release of this new species was, in particular, evident in the adsorption processes, for which the processing time, i.e., interaction time between cobalt and surface coating, was higher than during filtration. These new species in solution were found to be oxy-sulfuric radicals generated by the interaction of cobalt ions with sulfur groups. Furthermore, it was shown that in the presence of other contaminants, such as MO, the interaction of sulfur groups with Co is preferred, as evidenced by the formation of oxy-sulfur radicals [140].

To conclude, this section has shown that the Nexar polymer acts as a smart surface coating of commercial hydrophobic filters, resulting in a hydrophilic, acidic, and negatively charged surface. Thanks to these properties, the coating showed antimicrobial and antibiofouling properties, and the as-prepared filter could be used for the selective adsorption/filtration of cationic contaminants. The addition of GO is shown to increase the absorption capabilities of the modified filter. The UV treatment of the surface coating not only increases the surface wettability but induces coating modifications (for example, electron enrichment) that make it reactive with water contaminants. Particularly interesting is the interaction of cobalt ions with sulphonic groups of the polymer that leads to the formation of secondary species (oxy-sulfur radicals). However, in the future, these radicals could be used as oxidizing species or active species to detect metals in liquids. Indeed, oxy-sulfur radicals have a higher oxidizing potential than hydroxyl radicals, and they could be used in photocatalytic processes to degrade organic contaminants. Furthermore, concentrations of Co ions in liquids lower than 17.5 mM would be hard to detect by UV-Vis absorption measurements, while the peak of the oxy-sulfur radical at 256 nm is well detectable. Consequently, it would be easier to detect the presence of oxy-sulfur radicals generated by the interaction of Co ions/sulphonic groups, and their amount could be related to the amount of cobalt ions.

## 5. Other Applications of Nexar^TM^

In addition to water purification, sulfonated pentablock copolymer has been recently investigated as a cheap and efficient alternative to well-known commercial materials in different applications, for example, as proton exchange membrane in fuel cells or water splitting electrolyzers, in systems for dehumidification or CO_2_ capture, or as a template for the self-assembling of nanoparticles. In this section, some examples of the use of Nexar in these types of applications are reported.

### 5.1. Nexar^TM^ in Energy Application

Sulfonated aromatic polymer membranes have been considered possible substituents for the most studied and used proton exchange membranes, i.e., Nafion^®^ membranes. Nafion is a perfluorinated sulfonic acid polymer that was widely used in many applications, such as fuel cells, electrochemical hydrogen compressors, ion-exchange resins and catalysts, water purification, and flow batteries. Nafion has shown good performance, but it has high costs, and its synthesis and degradation generate toxic fluorinated by-products. High ionic conductivity and substantially high thermal stability make sulfonated aromatic polymers promising membranes that can operate at high temperatures. Furthermore, the preparation of these membranes is also economical and relatively easy, and no toxic fluorinated by-products are generated.

In this field, Nexar^TM^ was studied as an alternative to Nafion for proton exchange membranes in fuel cells because of its higher conductivity and lower activation energy with respect to Nafion [146,147,148]. Hwang et al. characterized a series of Nexar films with different ion exchange capacities (1.0, 1.5, and 2.0 meq g^−1^) and investigated them as ionomers in hydrogen fuel cells [149]. Compared with Nafion, all Nexar films exhibited higher proton conductivity (>0.2 S cm^−1^ at 80 °C, 90% RH) and higher fuel cell performance at 50 °C, 100% RH. In contrast, at higher operating temperatures (80 °C, 100% RH), the fuel cell performances were found to be better for Nafion than for Nexar membranes.

In order to further increase the ion conductivity and thermal stability of Nexar films for proton exchange membranes, composites with silica nanoparticles or graphene oxide flakes were prepared [146,147,148]. Incorporating silica and sulfonated silica nanoparticles into Nexar^TM^ drastically changed the water uptake and swelling ratio. In particular, the addition of sulfonated silica nanoparticles improved proton conductivity by a 58.8 factor compared with pristine Nexar. Similarly, modified Nexar membranes produced by the addition of GO and sulfonated GO flakes were tested as proton exchange membranes in fuel cells [146,147,148]. Nexar-GO membranes showed higher water uptake and higher swelling than pristine Nexar, whereas Nexar-GO-SO_3_H indicated marginally lower values. The highest proton conductivity was measured for Nexar-GO-SO_3_H, suggesting this membrane is a potential material for PEM fuel cell membranes.

Another interesting characteristic of Nexar polymer is the possibility of tuning its structure, for example, by changing the dispersing medium. Ionomer composition and morphology impact functional group distribution, water and ion-transport, and physical properties related to toughness and degradation resistance. Huang prepared Nexar proton exchange membranes by casting from apolar and polar solvent solutions, and the effect of film morphology on conduction properties was investigated [150]. The film cast from apolar solvent showed a random distribution of discrete sulfonated domains, while an ordered s-PBC morphology consisting of lamella and hexagonally packed ion groups was achieved in polar solvent. The last morphology positively affected conductivity, which increased from 4.5 mS/cm for the film prepared in an apolar solvent to 47.8 mS/cm for the one prepared in a polar solvent. Similarly, the latter showed improved fuel cell power (160 mW/cm^2^ versus 19 mW/cm^2^).

Filice et al. tested Nexar film as a low-cost and efficient alternative to Nafion as a proton exchange membrane in the PEM water electrolyzer [93]. As reported, Nexar showed a water uptake one order of magnitude higher than Nafion (168% versus 25%, respectively) as a consequence of the higher density of sulfonic groups. This resulted in an increased proton conductivity of about four times with respect to Nafion: from the impedance curves recorded at 0 V bias, Nexar^TM^ showed a resistance value one order of magnitude lower than for Nafion, i.e., 0.38 Ω instead of 1.55 Ω. Furthermore, high water uptake permits the system to remain hydrated and favors the occurrence of electrochemical reactions. This explains why Nexar’s performance is better compared with Nafion in the same working conditions. The reported values by the authors were obtained for membranes without activation processes and for low humidity content so that higher values could be expected in the case of fully hydrated membranes.

Thanks to its high sulfonic group density and mechanical/thermal stability, Nexar has shown good results as proton exchange membranes in fuel cell or water splitting applications, being an efficient and low-cost alternative to Nafion. It is likely that these results could be further ameliorated by the addition of suitable nanoparticles or by changing the experimental conditions (i.e., solvent) during Nexar film preparation.

### 5.2. Nexar^TM^ for CO_2_ Capture and Dehumidification

Nexar films have been investigated as separation membranes in gas and liquid media. Fan et al. studied sulfonated and unsulfonated pentablock copolymer ionomers with respect to gas (CO_2_, O_2_, and CH_4_), liquid, and ion transport in order to evaluate their potential application as a polymer electrolyte membrane [97,151]. They observed that gas transport properties were slightly dependent on the sulfonation degree, so gas permeability depended more on the unsulfonated domains. Sulfonation increases the gas solubility and transport. In particular, CO_2_ had a higher solubility, suggesting a greater affinity with the material. Water uptake, methanol permeability, and proton conductivity increased with the sulfonation degree. The higher water content within sulfonated PBC ionomers resulted in swelling that improved methanol transport because sulfonated domains become increasingly connected.

Dai et al. evaluated membrane gas-separation performance and molecular transport of CO_2_ through membranes prepared by incorporating 1-butyl-3-methylimidazolium tetrafluoroborate ([Bmim][BF_4_]) ionic liquid (IL) into a Nexar^TM^ film and these membranes were evaluated at different relative humidities [152]. The incorporation of IL into Nexar noticeably improved its thermal stability and promoted swelling and ordering. All induced modifications resulted in enhancing CO_2_ permeability through membranes in the dry state. A maximum permeability of 194 Barrers and a maximum CO_2_/N_2_ selectivity of 128 were observed after the introduction of water vapor into the gas.

Air conditioning is one of the essential requirements for households and workstations. The highest energy-consuming component in air conditioning is dehumidification, which occurs through membranes. Nexar^TM^ was a promising alternative to Nafion as a cost-effective and energy-efficient membrane in proton exchange membrane electrolytic dehumidification [153]. Nexar membranes at two different ion exchange capacities were evaluated for proton exchange membrane (PEM) electrolytic air dehumidification. Water vapor transmission rate, water removal energy efficiency, and electrochemical analysis were performed under various humidity conditions, and potential directions were applied. Compared with Nafion, Nexar exhibited a higher water removal energy efficiency and higher water vapor transmission rate but lower energy efficiency because of the higher water sorption.

Nexar^TM^ was also used as a coating in commercial polyetherimide hollow fiber support for the separation of water vapor from humidified air [154]. The coating was prepared in a polar solvent in order to form a lamellar/parallel cylindrical structure separated equidistantly within the film, which ensured higher water vapor transfer efficiency. The membrane showed water vapor permeance up to 9089 GPU with water vapor to nitrogen selectivity up to 3870. The membrane reduced the relative humidity from 80% to 41%, proving it is one of the competitive materials for membrane dehumidification.

These results confirm that Nexar could also be used as an efficient and low-cost material in gas separation applications such as dehumidification and the selective removal, and subsequent capture, of CO_2_ from mixed gas streams to reduce the environmental contamination largely responsible for global climate change.

## 6. Conclusions and Future Perspectives 

In this Review, we provided an overview of the use of a sulfonated pentablock copolymers (s-PBC, commercial name Nexar^TM^, as itself or as a matrix for embedded nanomaterials (nanocomposites) for water remediation processes and other applications. We discussed several aspects related to the preparation and the use of Nexar and Nexar-based nanocomposites, which can be summarized as follows:

(a) Contaminant removal efficiencies, for practical applications, are strictly dependent on the polymeric structure. A good tradeoff between hydrophilicity and hydrophobicity is fundamental to obtaining good performance in filtration processes or ameliorating transport properties. For this scope, the structure of s-PBC, being formed by a hydrophobic backbone with hydrophilic functionalizations, plays a key role. Indeed, this molecular architecture is characterized by high hydrophilicity and high mechanical and thermal stability. Furthermore, its performance in terms of water uptake and transport is also affected by this architecture. Due to the presence of different polymeric units, it is formed by micellar structure affected by experimental parameters: its structure at nanolevel could be tailored by choice of the dispersing medium affecting its water uptake and transport properties. Taking advantage of these properties, Nexar^TM^ showed high performance in desalination, pervaporation, and filtration processes.
(b) Another advantage of this polymer is represented by the presence of sulfonilic groups on polymeric chains. Indeed, with respect to unsulfonated polymers, the presence of sulfonic groups on the polymeric backbone confers polymer-specific properties, such as higher hydrophilicity and acidic character that favors antimicrobial and antifouling activities, and the presence of active sites useful for selective adsorption of contaminants. These properties make Nexar^TM^ a good candidate for water remediation applications.
(c) Hybrid nanocomposite polymeric membranes could be easily prepared using the solvent casting method as free-standing films/membranes or as filter coating in adsorption, filtration, and photocatalytic processes for water purification. Embedded nanoparticles (graphene oxide, metal oxides) not only enhance the adsorption and filtration properties of Nexar itself, but they confer new functionalities such as photodegradation. Nexar acted both as a scaffold for photocatalytic NPs, avoiding their dispersion in the environment, and as an enhancer of the photocatalytic process. The main advantage of polymeric films is that they can be easily managed, regenerated, and reused without any release of nanomaterials into the environment. In particular, the Nexar properties and the reasons for its use were described, and several examples of its application for the removal of contaminants (i.e., microorganisms, organic molecules, and heavy metals) by different methodologies (such as adsorption, filtration, and photocatalysis processes) were reported.
(d) The molecular architecture of Nexar^TM^ is also responsible for the excellent transport properties of these materials, making it a good candidate for other applications such as devices for gas transport (i.e., filters, CO_2_ capture, and dehumidification) or as a proton exchange membrane in electrochemical devices for energy conversion and in this case it showed better performance than the well-known Nafion.

In the field of water purification, new interesting perspectives arise considering the high reactivity of sulfonilic groups towards external agents, such as UV irradiation treatments, that, as shown above, induce surface modifications (surface wettability increase, electron charge enrichment, and higher reactivity towards water contaminants such as MB and Co ions) favoring both the detection and removal of pollutants. Sulfonilic groups, indeed, could be used for the formation of secondary species (such as oxy-sulfur radicals) that are highly active in the degradation of contaminants.

Nexar’s antibacterial and antibiofouling activity without the use of chemicals and the production of toxic by-products are also interesting novelty properties of this polymer that are revealed to be suitable for application in harsh environments (high acidity or high salinity).

The last but not least important aspect of this polymer is the fact that it is suitable for other technological applications, from filters to electrochemical cells, hydrogen pumping, dehumidification, etc., requiring high transport properties and chemical, thermal, and mechanical stability, opening the route to the development of a system combining water/air purification with detection and/or energy conversion.

## Figures and Tables

**Table 1:** Different water pollutants, sources, their effects, and remediation strategies.

Pollutants	Sources	Effects	Remediations
Organic pollutants	Natural organic matter, Industrial waste (dyes, pesticides, chlorinated compounds, pharmaceuticals)	Mutagenicity, pH, COD,	Coagulation, membrane filtration, ion exchange/adsorption, ozonation/biodegradation, UV/Vis photocatalysis
Inorganic pollutants	Soil-erosion, power plants (Metals/Metalloids, nitrates, phosphates)	Acidity, hardness,	Adsorption, chemical precipitation, coagulation, flocculation, ion exchange, and membrane filtration
Microorganisms	Sewage, animal excrement (*E. coli*, *Bacillus subtilis*, *Pseudomonas aeruginosa*, *Enterococcus faecalis*, *Giardia lamblia*)	Waterborne disease	Halogenated compounds Disinfection compounds UV light UV/Vis photocatalysis
Emerging contaminants	Synthetic Natural New pathogens	Deleterious effects on endocrine systems and thyroid gland, infertility, cancer	Coagulation Flocculation membrane technology adsorption UV/Vis photocatalysis Biological treatment

**Table 2:** Removal ability by adsorption and photocatalysis of different Nexar and Nafion nanocomposites towards MB and MO.

Membrane	Removal Ability by Adsorption (3 h) * MO Peak Shift (pH Change)		Removal Ability by Photocatalysis (UVA-Blue Light, 3 h)	
	MB (C_0_ = 1.5 × 10^−5^ M)	MO (C_0_ = 2 × 10^−5^ M)	MB (C_0_ = 1.5 × 10^−5^ M)	MO (C_0_ = 2 × 10^−5^ M)
Nafion	≈70%	≈35–40% * (pH = 6 to 3.7)	≈82%	47%
Nafion-TiO_2_	≈60%	≈30% * (pH = 6 to 3.7)	≈70%	67% *(TiO_2_ 70mg/L, w/o Nafion: 72%)*
*Nafion-GO*	*80%*	≈35% * *(pH = 6* *à* *3.7)*	*≈92%*	*46%*
*Nexar*	*≈94%*	*≈20% (pH = 2)* *(no removal @pH = 6)*	*≈90%*	*≈29%*
*Nexar-TiO_2_*	*≈92%*	*≈29% (pH = 2)*	*≈90%*	*≈71% (pH = 2)* *(≈20% @pH = 6)*
*Nexar-GO*	*≈87%*	*≈48% (pH = 2)* *(no removal @pH = 6)*	*≈92%*	*≈70% (pH = 2)* *(No removal @pH = 6)*

**Table 3:** Q_t_ values for the adsorption of Fe^3+^ ions after 15 and 180 min, respectively, for all the investigated coating layers on the PP filter.

Coating Layer	Q_t_ of Fe^3+^ Ions (mg/g)	
	15 min	180 min
No coating (only PP)	0.269	1.237
s-PBC/PP	1.019	3.124
s-PBC-GO 0.17%/PP	6.277	2.939
s-PBC-GO 0.34%/PP	6.096	3.261
s-PBC-GO 0.67%/PP	5.990	1.511

**Table 4:** Q_t_ values for all the samples used in filtration and adsorption processes of Fe^3+^ and Co^2+^ ions in water solutions reported by [139,140].

Filter	Q_t_ of Fe^3+^ Ions (mg/g)		Q_t_ of Co^2+^ Ions (mg/g)	
	Adsorption (180 min)	Filtration	Adsorption (180 min)	Filtration
PP	0	0.30	0	5.46
s-PBC@PP	7.75	4.79	21	24
s-PBCGO@PP	5.86	5.10	37	21
